# The role of m6A epigenetic modifications in tumor coding and non-coding RNA processing

**DOI:** 10.1186/s12964-023-01385-w

**Published:** 2023-12-15

**Authors:** Tongxuan Wen, Tong Li, Yeqiu Xu, Yuanzhuang Zhang, Hai Pan, Yong Wang

**Affiliations:** 1 Department of Neurosurgery, Central Hospital Affiliated to Shenyang Medical College, Shenyang, Liaoning 110024 P.R. China; 2 Fourth Department of Orthopedic Surgery, Central Hospital Affiliated to Shenyang Medical College, Shenyang, Liaoning 110024 P.R. China

**Keywords:** m6A modification, RNA methylation, Epigenetics, Immunotherapy, mRNA processing, Non-coding RNA, Tumors

## Abstract

**Background:**

Epigenetic modifications of RNA significantly contribute to the regulatory processes in tumors and have, thus, received considerable attention. The m6A modification, known as N6-methyladenosine, is the predominant epigenetic alteration found in both eukaryotic mRNAs and ncRNAs.

**Main body:**

m6A methylation modifications are dynamically reversible and are catalyzed, removed, and recognized by the complex of m6A methyltransferase (MTases), m6A demethylase, and m6A methyl recognition proteins (MRPs). Published evidence suggests that dysregulated m6A modification results in abnormal biological behavior of mature mRNA, leading to a variety of abnormal physiological processes, with profound implications for tumor development in particular.

**Conclusion:**

Abnormal RNA processing due to dysregulation of m6A modification plays an important role in tumor pathogenesis and potential mechanisms of action. In this review, we comprehensively explored the mechanisms by which m6A modification regulates mRNA and ncRNA processing, focusing on their roles in tumors, and aiming to understand the important regulatory function of m6A modification, a key RNA epigenetic modification, in tumor cells, with a view to providing theoretical support for tumor diagnosis and treatment.

Video Abstract

**Supplementary Information:**

The online version contains supplementary material available at 10.1186/s12964-023-01385-w.

## Background

Epigenetics investigates alterations of gene transcription that are heritable, independent of the changes in gene sequence [1], including chemical modifications [2] such as DNA methylation [3], RNA methylation [4], histone modifications [5], and chromatin conformational changes [6]. In the realm of epigenetics, research on RNA methylation has gained significant attention. The RNA modification by methylation includes N6-methyladenosine (m6A) [7], N1-methyladenosine [8, 9], and 5-methylcytosine [10]. m6A is an extremely important internal modification in non-coding RNAs (ncRNAs) and messenger RNA (mRNA) and is common in prokaryotes and eukaryotes [11]. m6A modifications are mainly located at the 3′ UTR and the stop codon [7, 12] and are localized in two slightly different shared motifs: RRACH [13, 14] and DRACH [15] (H = U, A, or C; R = A or G; D = U, A, or G), which assume crucial roles in multiple RNA metabolic processes, such as miRNA processing and maturation, mRNA splicing, and lncRNA-mediated transcriptional repression [16, 17]. Furthermore, RNA epigenetic modifications mediated by m6A modifications are essential in physiological activities, such as control of the biological clock [18], sperm production [19], embryo development [20], maintenance of embryonic stem cell pluripotency [21], T cell homeostasis [22], heat shock response [23], and regulation of cardiac contractile function [24]. As mature mRNA control is closely linked to human illness, m6A modifications have been demonstrated to be related to many disorders, such as obesity [25], type II diabetes [26], infertility [27], and neuronal diseases [28]. One of the topical areas of research remains the association between m6A modifications and tumors. The impact of m6A modifications on malignant biological behaviors (e.g. tumor proliferation, invasion, metastasis, stemness maintenance, and drug resistance) is based on their modulation of mature mRNA processing [29–33]. The mutual regulation between m6A modifications and ncRNAs also plays an important role in tumor pathogenesis and potential mechanisms of action [4, 34]. Currently, more and more studies are revealing that dysregulation of m6A modification is closely related to cancer onset, progression, aberrant energy metabolism, radiotherapy resistance, and immune evasion, as well as cancer stem cell self-renewal and the tumor microenvironment [4, 35].

### Composition of m6A

**Fig. 1:**
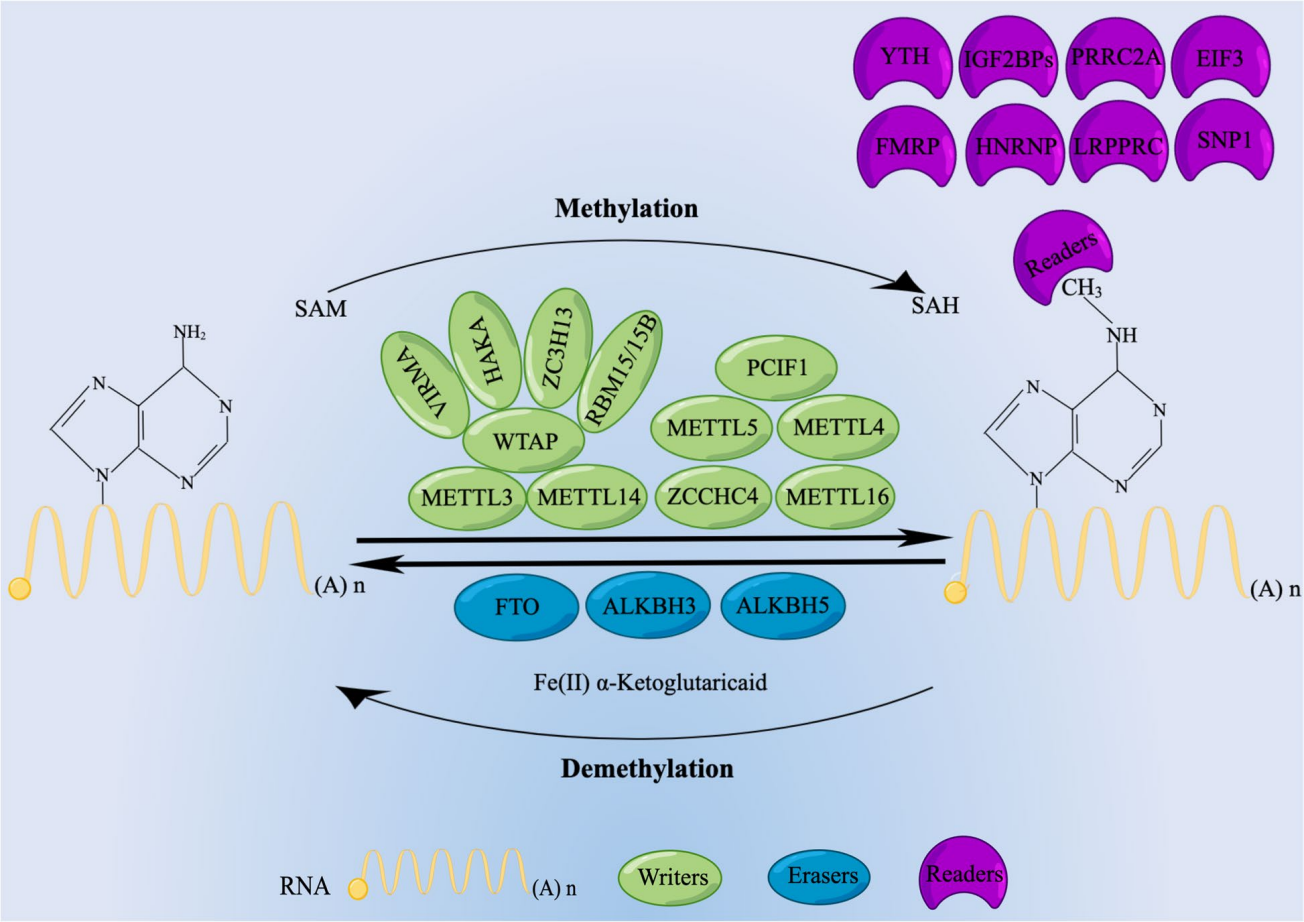
Classification chart for m6A regulators. The m6A modification is a dynamic and reversible epigenetic modification regulated by the “author” and “eraser” and is mainly catalyzed by the m6A methyltransferase complex, which includes the major components METTL3/METTL14/WTAP and other regulatory proteins (RBM15/15B, ZC3H13, HAKA, and VIRMA). In addition, METTL16, METTL5, METTL4, ZCCHC4, and PCIF1 are methyltransferases that directly catalyze m6A modifications in RNA molecules. The eraser consists mainly of FTO, ALKBH3, and ALKBH5. The “readers” are binding proteins that recognize the m6A modification and determine the fate of target RNAs, and they mainly include members of the YTH domain-containing family, the IGF2BP family, the HNRNP family, eIF3, PRRC2A, and FMRP

Investigations into the proteins associated with m6A have revealed that m6A methylation is an actively modifiable process controlled by the MTases complex (writer), demethylases (erasers), and MRPs (readers) [36] (Fig. 1).

### m6A writers

Writers "write" methylation modifications to RNA, i.e., they mediate the processes of methylated modifications to RNA. At least seven components make up this complex: zinc-finger CCCH-type containing 13 (ZC3H13), CBL proto-oncogene-like 1 (CBLL1; also known as HAKAI), vir-like m6A MTases-associated protein (VIRMA/KIAA14), RNA-binding motif protein 15/15B (RBM15/15B), WT1-associated protein (WTAP), and MTases-like 3/14 (METTL3/14). METTL14 and METTL3 are the predominant molecules among them, responsible for catalyzing mRNA methylation of m6A (as well as other cellular nuclear RNA) both in vivo and in vitro [37, 38], and WTAP is another key component of this MTases complex [39]. Functioning as the catalytic core, the methyl group can be transferred to the adenine portion of the receptor from SAM by METTL3. As a platform for RNA binding, METTL14 can facilitate RNA substrate binding and stabilizing of the complexes. Deposition of m6A on nuclear RNA is induced by dimerization of the METTL3-14 complex, while WTAP interaction with the heterogeneous complexity influences the activities of m6A MTases and the accurate location of methylation sites in vivo [40]. RBM15 helps recruit complexes to their target sites. VIRMA is engaged in site-specific METTL3-METTL14-WTAP recruitment, and HAKAI is an important component of MTases [41]. In the nucleus, ZC3H13 acts as an anchor for WTAP, Virilizer, and Hakai to facilitate m6A methylation [42]. In addition, METTL16 has been reported to modify the methyl transfer of mRNAs and U6-snRNAs [43, 44]. METTL5 and ZCCHC4 are formyltransferases that intermediate the m6A modifications of human 28S and 18S rRNAs, respectively [45]. A recent study has shown that the methylation of m6A of 2-O-methylated adenine at the 5′ end of mature mRNA is catalyzed by phosphorylated CTD-interacting factor 1 (PCIF1) [46]. m6A methylation of U2 snRNA is catalyzed by METTL4 and participates in pre-mRNA splicing [47].

### m6A erasers

Erasers can “erase” the signal associated with the methylation modification of RNA to mediate the process of RNA demethylation. The removal of m6A from RNA is currently known to be catalyzed by demethylation of AlkB homolog 3/5 RNA demethylase (ALKBH3/5), obesity-associated protein (FTO), or Fat mass [36, 48]. FTO proteins belong to the non-heme Fe (II)/dioxygenases AlkB family, which are α-ketoglutarate- and Fe(ii)-dependent; in addition, FTO can oxidize N-methyl to hydroxymethyl at the m6A site using α-ketoglutarate and ferrous iron as the co-substrate and cofactor, respectively. Suppression or enhancement of both FTO and ALKBH3/5 alters the level of m6 in cells [27, 36, 49].

### m6A readers

Readers have the ability to detect RNA methylation modifications, which are instrumental in the translation and degradation of RNA downstream, thereby giving rise to various biological phenotypes. The reader "reads" in two modes: direct and indirect reading. Direct reading refers to selective binding to the RNA m6A region. The main proteins include the eukaryotic initiation factor 3 (EIF3), insulin-like growth factor 2 mRNA-binding proteins (IGF2BPs, including IGF2BP1/3), and the YTH structural domain family (YTHDC1/2 and YTHDF1/2/3). Among them, IGF2BPs recognize the common sequence GG(m6A)C under normal conditions and thus target mRNA transcripts to promote mRNA stability and translation; under stress conditions, they mediate the storage of target mRNAs by translocating stress granules [50]. Proline rich coiled-coil 2 A (PRRC2A) [51], leucine-rich pentatricopeptide repeat containing (LRPPRC) protein [52], fragile X mental retardation protein (FMRP) [53], and secondary wall-associated NAC domain protein 1 (SND1) [54] can maintain the stability of target mRNAs by reading m6A modifications. In indirect reading, m6A modifications alter the secondary structure of RNA; for example, heteronuclear RNA protein families (HNRNPA2B1, HNRNPC, and HNRNPG) [55].

### Function of m6A

m6A is mainly added to RNA by “writers” in the nucleus and is then recognized by “readers” in the nucleus or cytoplasm and influences the processing of RNA, including RNA processing and maturation, translation and degradation. Finally, the procedure of m6A modification becomes dynamic and reversible through the action of “erasers” in the nucleus or cytoplasm, thus acting to regulate a wide range of gene expression.

### m6A regulates mRNA processing

**Fig. 2:**
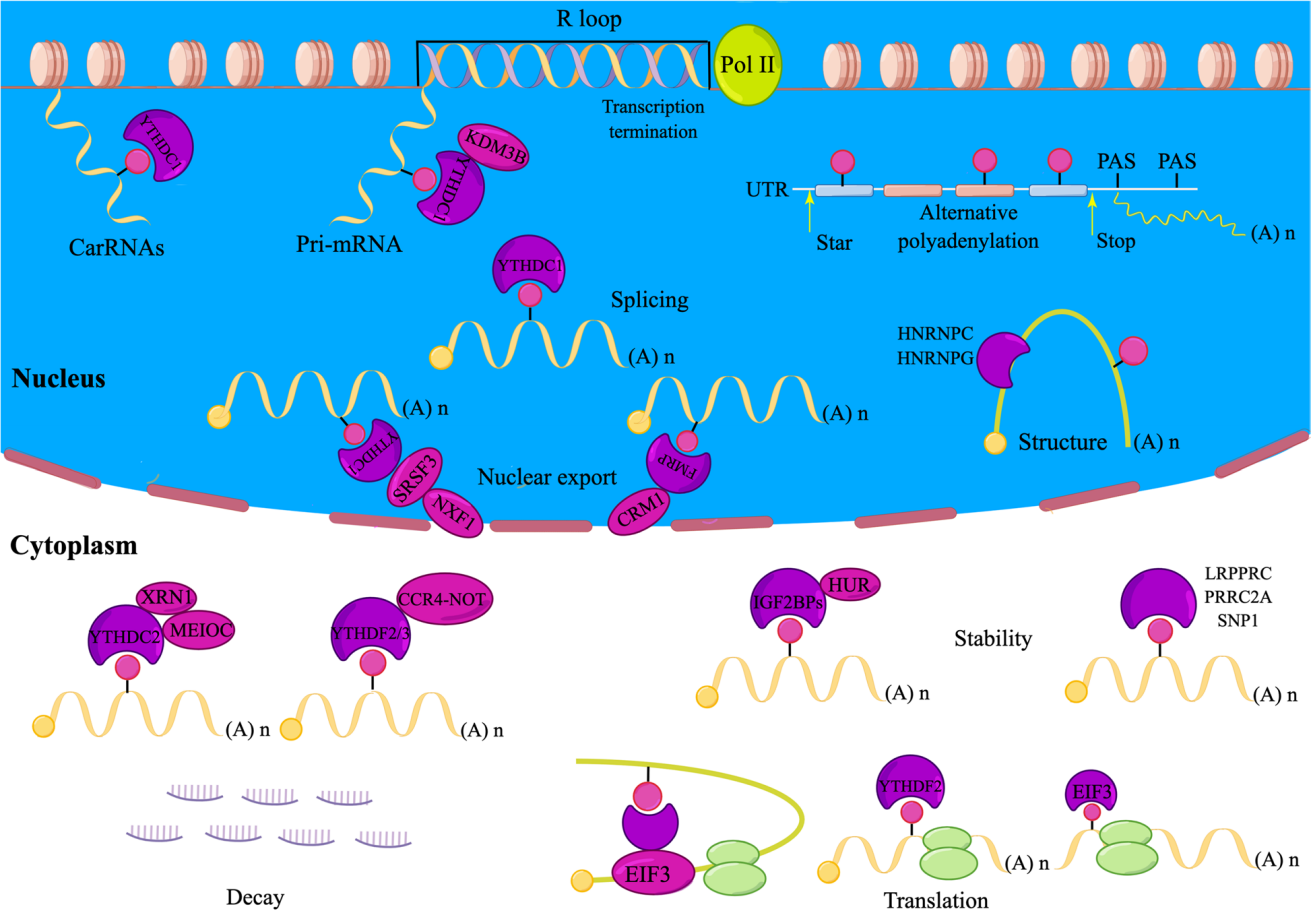
The processing of mRNAs is closely related to m6A modification. m6A regulators are involved in mRNA processing, including transcription, polyadenylation, shear and structure, nucleation, stabilization and degradation, and translation. Deregulation of m6A modification will lead to different fates of mRNAs

The complete processing of mRNA is closely connected to the modification of m6A. By binding directly or indirectly to RNA-binding proteins, m6A modifications are involved in mRNA maturation, organismal processing, extranuclear transport, translation, and RNA stabilization and are intimately linked to tumorigenesis and progression. Herein, we provide an overview about m6A in the processing of mRNA (Fig. 2 and Table 1).

**Table 1:** The role of m6a modifications in messenger RNA processing

RNA processing	m6A regulator	Mechanism	Function	References
Transcription	METTL3	METTL3-induced m6A modification promotes the formation of an R-loop around the transcription termination site of the m6A + gene, thereby reducing the read-through activity of Pol II	Promotes transcriptional termination	[58]
	YTHDC1	mediates degradation of METTL3-added m6A-modified CarRNA decay	Inhibits transcription	[57]
	YTHDC1	causes changes in chromosome structure by recruiting KDM3B, thereby removing H3K9me2	Transcription promotion	[56]
Polyadenylation	METTL3	m6A-containing transcripts tend to use the proximal APA site (proximal, rather than the transcription start site), resulting in a shorter 3′ UTR	Regulation of alternative polyadenylation	[59, 60]
splicing and structure	YTHDC1	Recruitment of SRSF3, exclusion of SRSF10	Regulating variable splicing	[66]
	HNRNPG	uses the Arg-Gly-Gly motif to bind directly to the carboxy-terminal structural domain (CTD) of RNAP II and interacts with phosphorylated CTD and m6A-modified nascent pre-mRNA or "m6a switch" mechanism	Regulating variable splicing	[67, 70]
	HNRNPC	"m6a switch" mechanism	Regulating variable splicing	[55, 69]
	ALKBH5	Promotes pre-mRNA variable splicing	Promotes melanoma tumor progression	[65]
	HNRNPC	promotes the selective splicing of TATA box-binding protein-associated factor 8 in an m6A-dependent manner	Promotes PDAC progression	[68]
Nuclear export	METTL3	Depletion of METTL3 inhibits nuclear export of TRAF6 mRNA	Suppression of nuclear output	[72]
	ALKBH5	Reduction of ALKBH5 leads to phosphorylation of ASF/SF2 and enhanced binding to the TAP-p15 complex	Promotes exportation	[27, 73]
	YTHDC1	Promotes NXF1-mediated nuclear export	Promotes exportation	[74]
	FMRP	preferentially recognises m6A-modified RNA and binds to CRM1	Promotes exportation	[75]
Stability	YTHDF2	Recruits CCR4-NOT complexes	Mediates RNA degradation	[78]
	YTHDF3	Cooperates with YTHDF2	Mediates RNA degradation	[79]
	YTHDC2	Cleavage helicase activity	Mediates RNA degradation	[83]
	IGF2BPs	Assisted by HUR, PABPC1, MATR3I, IGF2BPs bind to target m6A sites via the KH structural domains	Stabilizing RNA	[50]
	FMRP	Interacts with YTHDF2	Stabilizing RNA	[53]
	LRPPRC	Formation of LRPPRC-SLIRP complexes	Enhances mitochondrial RNA stability	[52]
	PRRC2A	stabilizes olig2 mRNA stability by recognizing GGACU sequences	Stabilizing RNA	[51]
	SND1	Stabilizes ORF50 mRNA in an m6a-dependent manner	Stabilizing RNA	[54]
	YTHDF2	Accelerates UBN1, LXRα77 and HIVEP278 mRNA degradation	promotes GBM progression	[80–82]
	IGF2BP2	enhances HMGA1 mRNA stability	promotes CRC progression	[86]
	FTO	mediates ASB2, RARA mRNA degradation	promotes AML progression	[88]
	FTO	increases M2F1 mRNA stability by decreasing m6a methylation levels	promotes progression of squamous carcinoma	[89]
	METTL3	enhances ZMYM1 expression by increasing m6A methylation levels in ZMYM1 mature mRNAs	promotes GC progression	[91]
	METTL3	causes SOCS2 mRNA degradation through the addition of m6A modifications	promotes HCC progression	[90]
Translation	METTL3	interacts with EIF3h to promote mRNA recycling	enhances translation and promotes lung cancer progression	[95]
	YTHDF1	promotes ribosome assembly of m6A-mRNAs by generating loop structures with EIF3 and EIF4G	enhances translation	[98]
	YTHDF3	assists YTHDF1 binding to ribosomal 40S/60S subunits	enhances translation	[99]
	EIF3	binds to mRNA containing m6A sites in the 5′ UTR to initiate cap-independent translation	enhances translation	[102]
	YTHDC2	promotes translation initiation of HIF-1 alpha by unravelling the 5′ UTR of mRNAs	Enhancing translation and Promotion of CRC metastasis	[103]
	IGF2BPs	bind translation to mRNA stability	Enhancing stability and translation	[50]
	METTL16	interacts with EIF3a, EIF3b	enhances translation and promotes HCC progression	[96]
	METTL3	promotes C-MYC, BCL2, PTEN mRNA translation	promotes AML progression	[92]
	METTL3	upregulates receptor tyrosine kinase AXL translation	promotes Ovarian cancer	[93]
	METTL3	induces GLUT1 translation in an m6a-dependent manner	promotes CRC progression	[94]
	ALKBH5	regulates energy metabolism by inducing GLUT1 translation in an m6a-dependent manner	promotes GBM progression	[97]
	YTHDF1	promotes translation of m6A-modified WNT6 and FZD9 mRNAs, leading to aberrant activation of their downstream signalling pathways	promotes CRC progression	[100]
	YTHDF2	enhances translation of OCT4 mRNA in an m6A-dependent manner	promotes HCC progression	[101]
	YTHDF3	Co-operates with YTHDF1 to enhance translation	promotes HCC progression	[79]

*PDAC* pancreatic ductal adenocarcinoma, *GBM* glioblastoma, *CRC* colorectal carcinoma, *AML* acute myeloid leukemia,*GC* gastric cancer, *HCC* Hepatocellular carcinoma

### Transcription

m6A, a recognized post-transcriptional modification, does not appear to be related to RNA transcription. However, studies have confirmed that YTHDC1 recruits lysine demethylase 3 B (KDM3B) to the corresponding chromatin region by recognizing m6A modifications in the mRNA, thereby removing the repressive histone H3 lysine 9 dimethylation (H3K9me2) tag and promoting transcription [56]. In addition, METTL3 has been shown to deposit the modifications of m6A on chromosome-associated regulatory RNAs (carRNAs), such as repetitive RNAs, enhancer RNAs, and promoter-associated RNAs, and YTHDC1 has the ability to trigger the degradation of a portion of these m6A-modified RNAs. Depletion of METTL3 or reduction of m6A modification levels using demethylase action in mouse models increases the carRNA levels, thereby promoting the transcription of downstream and the accessibility of chromatin [57]; METTL3-induced m6A modification also facilitates the R-loop formation around the transcription end site for m6A-containing protein-encoding transcripts (m6A + genes) to reduce the read-through activity of Pol II, leading to transcription termination [58].

### Polyadenylation

Most eukaryotic genes have multiple polyadenylation sites (PAS) that allow pre-mRNA to be polyadenylated at different sites; that is, alternative polyadenylation (APA). In cells with RNAi silencing of m6A MTases (METTL3, METTL14, and WTAP), there is a change in the propensity to select APA sites [59]. Further studies have shown that m6A-containing transcripts tend to use the proximal APA site (proximal, as opposed to the transcription start site) and thus have a shorter 3′ UTR [60]. As a critical molecular machinery, APA is engaged in various gene regulatory processes, such as mRNA maturation, mRNA stabilization, mRNA decay, and protein diversification [61], leading to reduced or increased expression of tumor suppressor genes or oncogenes, thereby promoting tumorigeneses and progression [62, 63]. However, the exact mechanism by which m6A regulates APA remains unclear.

### Splicing and structure

There is an important intrinsic link from m6A to selective splicing of mRNA. For example, analysis of m6A-IP/RNA-seq data from HepG2 cells with METTL3 deficiency showed differential expression of many genes which were methylated at the heterodimeric level, and that spliced exons and introns were significantly enriched for m6A peaks [64]. ALKBH5 was reported to promote pre-mRNA shearing, and knockdown of this gene significantly inhibited the growth of swollen melanomas and improved survival in mice during immunotherapy [65]. In addition, YTHDC1 recognizes m6A modifications, then promotes exon incorporation, and regulates the splicing of pre-mRNA by recruiting serine/arginine-rich splicing factor 3 (SRSF3) and rejecting serine/arginine-rich splicing factor (SRSF10) [66]. HNRNPG uses the Arg-Gly-Gly (RGG) motif to bind directly to the carboxyl-terminal domain (CTD) of RNA polymerase II (RNAP II) and regulates selective splicing by interacting with the phosphorylated CTD and m6A-modified nascent pre-mRNA [67]. In pancreatic ductal adenocarcinoma (PDAC), HNRNPC regulates the selective splicing of TATA box-binding protein-associated factor 8 (TAF8) in an m6A-dependent manner, thereby promoting tumor progression [68]. In addition, the formation of mRNA local structure and structure-specific RNA-binding proteins (RBP) function are closely connected to the modification of m6A. For example, HNRNPC is a nuclear-localized RBP with a tendency to sequentially bind single-stranded RNA to uracil. The modification of m6A weakens the hydrogen bonding between adenine and uracil and alters the structure of the RNA, exposing the polyuracil-binding sequence and facilitating HNRNPC binding [69]. In vitro gel electrophoresis migration assays demonstrated that m6A facilitated the binding of HNRNPC proteins. Reducing intracellular METTL3/METTL14 expression reduces the ability of HNRNPC to bind substrates, thus affecting the variable shear of mRNA [55]. Similarly, m6A-dependent changes in RNA structure could facilitate direct binding of m6A modified pre-mRNA to the low-complexity areas of HNRNPG [70]. This suggests that in addition to directly acting as a tag for RNA-binding proteins, m6A can act as anmature mRNA structural “switch” to indirectly regulate RNA-RBP interactions [55].

### Nuclear export

After RNA splicing, the translocation of maturing mRNA to the cytoplasm for translation or degradation occurs. The RNA nuclear egress is significantly connected to the modifications of m6A. Camper et al. demonstrated a significant delay in the nuclear export of mature mRNA after reduction of m6A in HeLa cells [71]. The depletion of METTL3 inhibits mRNA export [72], whereas reduced ALKBH5 causes ASF/SF2 phosphorylation, and enhances binding to the TAP-p15 complex, resulting in accelerated nuclear mRNA export [27, 73]. Additionally, by facilitating the attachment of RNA to the export bridging protein SRSF3 and nuclear RNA export factor 1 (NXF1), YTHDC1 could promote m6A-mediated nuclear export [74]. FMRP preferentially binds to m6A-modified RNA and facilitates nuclear export of target RNA through CRM1 [75]. Notably, even without m6A modifications, certain mature mRNA can exit the nucleus, suggesting that m6A modification may only act as a regulator of nuclear export, rather than a necessary translocation regulator [76].

### Stability

Stable mature mRNA levels are essential for gene expression, and the rate of degradation is a primary factor influencing cellular mature mRNA abundance. Moreover, m6A is a double-edged sword in terms of regulating mRNA stability. For example, in the cytoplasm, the YTHDF2 YTH structural domain could recognize the mRNA modified by m6A, whereas the functional domain at the other end mediates degradation of the YTHDF2-mRNA complex [77]. By identifying m6A and recruiting the CCR4-NOT deadenylase complex, YTHDF2 can promote the decay of mRNA [78]. Synergistic effects between YTHDF proteins have been reported, with YTHDF3 synergistically promoting mRNA degradation along with YTHDF2 [79]. In gliomas, YTHDF2 overexpression may simultaneously accelerate the degradation of UBXN1 [80], LXRα [81], and HIVEP2 [82] mature mRNA and promote malignant progression of the tumor. Additionally, YTHDC2 exhibits mature mRNA-unwinding helicase activity [83] and has an essential role in germ cell meiosis [84, 85]. In contrast, by binding to the target m6A site through the KH domain with the assistance of ELAV-like RNA-binding protein 1 (ELAVL1), IGF2BP1/2/3 can stabilize mature mRNA [50]. Additionally, IGF2BP2 could stabilize the mRNA of HMGA1 and protein expression, promoting colorectal cancer progression [86]. Notably, YTHDC2 and IGF2BP1/2/3 are able to combine and regulate mature mRNA stability through active translation [50, 87]. Proline-rich discoid coil 2A (PRRC2A) could potentiate mature mRNA stabilization depending on the presence of m6A by binding to the GGACU pattern shared in the coding sequence (CDS) [51]. In addition, FTO can destabilize mature mRNA and promote acute myeloid leukemia (AML) progression by reducing the m6A abundance of *RARA* and *ASB2* mRNA transcripts [88], as well as increasing mRNA stability by downregulating m6A in the transcripts of MZF1, resulting in the progression and metastasis of lung squamous carcinoma cells [89]. METTL3 promotes hepatocellular carcinoma proliferation and metastasis by promoting m6A modification at the *SOCS2* mRNA 3′ end, resulting in accelerated *SOCS2* mRNA degradation [90]. In contrast, METTL3 overexpression elevates m6A methylation in *ZMYM1* mature mRNA, stabilizes *ZMYM1* mature mRNA, and enhances its protein expression level, thus promoting invasive metastasis of gastric cancer [91]. In summary, these findings show that m6A modifications can impact mature mRNA stability and thus regulate tumor development.

### Translation

Most modifications of m6A occur in exons, and m6A remains in the spliced and maturing mRNA. Therefore, m6A modifications are also capable of regulating mRNA translation using many patterns. Depending on the presence of m6A modifications, the translation of the target gene mRNA can be promoted by METTL3, thus leading to malignant cancer development, including AML [92], ovarian cancer [93], and colorectal cancer (CRC) [94]. It can also promote lung cancer progression by directly recruiting translation initiation factors to increase RNA translation and by exerting a regulatory role independent of MTase activity [95]. Similarly, METTL16 regulates translation in hepatocellular carcinoma cells in an MTase activity-dependent and non-dependent manner in the nucleus and cytoplasm, respectively [96]. In addition, ALKBH5 catalyzes the *G6PD* mRNA demethylation and promotes G6PD translation, thus participating in glioma cells’metabolism of energy [97]. Of concern, YTHDF proteins have an important role in regulating protein translation, with YTHDF1 promoting ribosomal assembly of m6A-mRNA by generating the ring structures with EIF3 and EIF4G, thereby facilitating translation initiation [98]. YTHDF3 and YTHDF1 act together to boost mRNA translation by engaging with the ribosomal 40S/60S subunits [99]. In tumors, YTHDF1 promotes the translation of m6A-modified Wnt family member 6 (*WNT6*) and frizzled class receptor 9 (*FZD9*) mRNAs, leading to aberrant activation of their downstream signaling pathways, which play a crucial role in CRC proliferation, invasion and metastasis [100]; YTHDF2 enhances the translation of *OCT4* (oncogene) mRNA in an m6A-dependent manner and promotes hepatocellular carcinoma progression [101]. YTHDF3 interacts with YTHDF1 to facilitate the translation of hepatocellular carcinoma-targeting mRNAs [79]. In addition, studies have confirmed that m6A sequences within the 5′ UTR can act as m6A-induced ribosome engagement sites (MIRES) to facilitate cap-independent translation of mRNAs. For example, mRNAs with m6A sites in the 5′ UTR can bind directly to EIF3 and recruit the 43S complex to initiate translation in a manner that bypasses the 5′ cap-binding protein [102]. YTHDC2 promotes translation initiation of hypoxia-inducible factor-1 alpha (HIF-1 alpha) leading to colon tumor metastasis by unraveling the 5′ UTR of the mRNA [103].

These reports suggest that m6A modification-mediated translation of mRNAs may be involved in tumor development and may provide new targets for tumor therapy and diagnosis.

### Interaction of m6A and ncRNAs

**Fig. 3:**
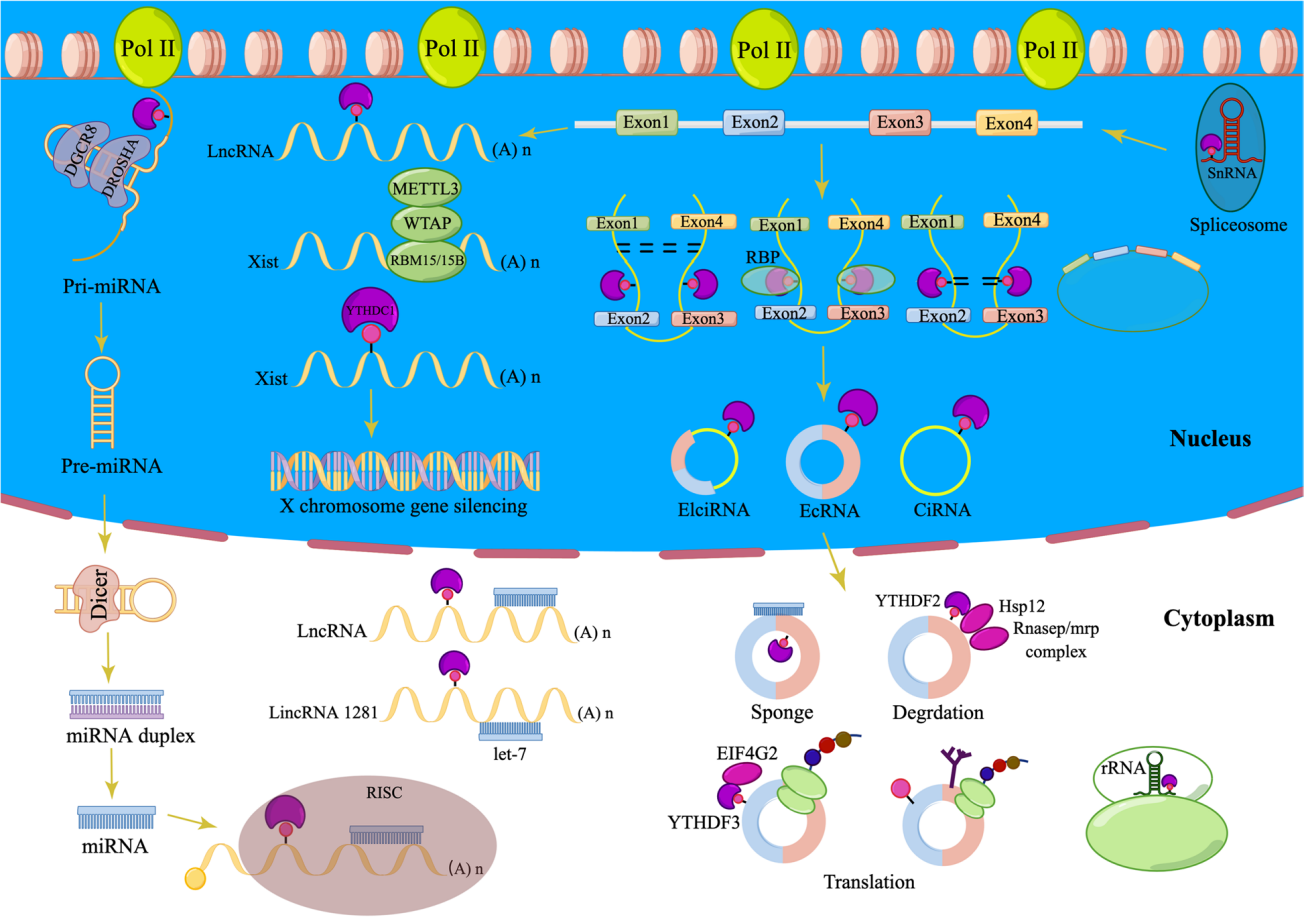
The effect of m6A modification on the processing of ncRNAs such as miRNAs, lncRNAs, circRNAs, etc. m6A regulatory factors are involved in multiple steps of ncRNA processing including splicing and structure, translation, and degradation. Notably, ncRNAs also have regulatory effects on m6A regulatory factors

The non-coding RNAs (ncRNAs) mainly include circRNAs, lncRNAs, and miRNAs [104], which are closely connected to the proliferative and invasive capacities and survival of tumor cells [105]. m6A is also found in several ncRNAs [15, 60]. m6A not only affects the degradation, transport, and shearing of ncRNAs [55, 70, 106, 107] but also participates in the functional regulation of various cells by controlling ncRNA expression, affecting the pathology of various diseases, including cancer [108, 109]. Notably, ncRNAs have also been found to regulate m6A modifications [110, 111]. This section summarizes the connections of m6A to the processing of ncRNA (Fig. 3 and Table 2).

**Table 2:** The role of m6a modification in non-coding RNA processing

RNA processing	m6A regulator	Mechanism	Function	References
miRNA processing	METTL3	promotes binding to HNRNPA2B1, DGCR8 and Dicer by methylating pri-miRNAs	promotes pri-miRNA processing	[117, 118]
	METTL3	promotes miR-221/miR-222 processing, leading to reduced PTEN expression	promotes Bca progression	[119]
	METTL3	enhances processing maturation of miR-1246 via m6a methylation	promotes CRC progression	[120]
	METTL14	METTL14-mediated methylation modification of m6a affects binding of pri-miR-126 to DGCR8	Inhibition of HCC growth	[121]
	ALKBH5	mediates miRNA demethylation by interacting with DDX3	Regulates cell growth and proliferation	[122]
	HNRNPC	Silencing of HNRNPC reduces miR-21 expression, thereby inhibiting the AKT-p70S6K pathway	Promote GBM cell migration and invasion	[123]
	NKAP	mediates the maturation of m6A-modified pri-miR-25, leading to a decrease in PHLPP2 expression, which promotes the activation of AKT signalling	promotes Pancreatic cancer progression	[124]
	TARBP2	Modifying dependencies via m6a	Regulation-miRNA processing maturation	[125–128]
	HNRNPA2B1	inhibits miR-29a-3p, miR-29b-3p and miR-222 expression	endocrine resistance in breast cancer cells	[129]
lncRNA structure	HNRNPC	"m6a switch" mechanism	Structural changes	[136]
lncRNA stability	METTL3	enhances MALAT1 stability	promotes IDH-wildtye glioma progression	[134]
	VIRMA	Enhances CCAT1, CCAT2 stability	ncreases Pca invasiveness	[135]
	METTL3	increases ABHD11-AS1 stability	promotes NSCLC progression	[136]
	METTL3	stabilizes FAM225 and initiates ceRNA model	promotes NPC progression	[138]
	METTL3	stabilizes RHPN1-AS1 and initiates ceRNA model	promotes ovarian cancer	[139]
	METTL3	stabilizes LINC00958 and initiates ceRNA model	promotes adipogenesis and progression of HCC	[140]
circRNA biosynthesis	METTL3	YTHDC1 direct the back-splicing reaction CircZNF609	Regulation of circRNA synthesis	[147]
	METTL3	installs m6a in the reverse complementary sequence of the circ1662 flanking intron to promote circRNA production	promotes CRC invasive metastasis	[148]
circRNA nuclear export	YTHDC1	enhances the stability of HMGA2 mRNA by generating the RNA–protein ternary complex circNSUN2/IGF2BP2/HMGA2	promotes colorectal cancer metastasis	[149]
	METTL14	Recognition of the GGACU structure promotes the export of m6A-modified circGFRα1	Promotes nuclear export	[150]
circRNA translation	METTL3	YTHDF3 cap-independent start translation	Driving translation	[147, 158]
	METTL3	Promotes circE7 translation	Promotes cervical cancer progression	[163]
circRNA stabilization	YTHDF2	formation of the YTHDF2-HRSP12-RNase/MRP complex	circRNA degradation	[152]
	METTL3	stabilizes circ0000069 and initiates ceRNA model	promotes cervical cancer progression	[155]

*BCa* bladder cancer, *CRC* colorectal carcinoma, *NSCLC* non-small-cell lung cancer, *HCC* Hepatocellular carcinoma, *NPC* nasopharyngeal carcinoma

### miRNAs

miRNAs, approximately 22 nucleotides in length, are ncRNAs that can regulate gene expression by generating an RNA-induced silencing complex (RISC) that interacts with the target mRNA 3′ UTR, leading to the degradation of mRNA and repression of translation [112, 113].

### m6A methylation promotes miRNA processing

m6A modification is an essential machinery in miRNA biosynthesis [106]. MiRNA maturation involves three steps: first, transcription, where pri-miRNA is formed in the nucleus; second, with the help of Drosha and DGCR8, pri⁃miRNA is converted to pre⁃miRNA; third, export of pre⁃miRNA from the nucleus and cleavage by Dicer to mature miRNA in cytoplasm [114]. In particular, DGCR8 initiates miRNA maturation by recognizing junctions between the stem and flanking single-stranded RNAs of pri-miRNA hairpins, which then recruit DROSHA. First, to generate a product of pre-miRNA, both strands near the base of the stem are cleaved by DROSHA [115, 116]. METTL3 can methylate the ⁃miRNA and thus mark the ⁃miRNA for recognition and processing by DGCR8 [117, 118]. In BCa, Han et al. showed that by interacting with DCGR8, miR-221/miR-222 maturation is positively affected by METTL3 overexpression, depending on the presence of m6A modifications, thereby reducing the expression of human chromosome 10 deletion phosphatase and genes of phosphate and tension homology deleted on chromosome 10 (PTEN), inhibiting the cancer-inhibiting effect of PTEN, and enhancing the proliferation of BCa cells [119]. In colon cancer, Peng et al. demonstrated that METTL3 can significantly enhance miR-1246 maturation by methylating miR⁃1246, leading to cancer cell migration and invasion [120]. METTL14 overexpression affects the binding of pri-miR-126 to DGCR8 depending on the presence of m6A modifications, thereby inhibiting HCC migration and invasion [121]. ALKBH5 mediates miRNA demethylation by interacting with DDX3 to regulate cell growth and proliferation [122]. Previous research has demonstrated that m6A reads are involved in the biogenesis of miRNAs. For example, HNRNPC can bind to pri-miR-21 directly, and silencing HNRNPC reduces miR-21 expression, thereby inhibiting the AKT-p70S6K pathway, leading to GBM cell migration and invasion [123]. NKAP mediates the maturation of m6A-modified pri-miR-25, leading to reduced PHLPP2 expression, thereby promoting AKT signaling activation and the development and progression [124]. Additionally, TAR RNA-binding protein 2 (TARBP2) has been reported to potentially regulate miRNA processes by mediating m6A modifications [125–128]. In summary, m6A modifications can affect the maturation of miRNAs, which in turn can regulate miRNA levels and influence tumor progression.

### m6A methylation suppresses miRNA processing

HNRNPA2B1 can interact with DGCR8 to promote miRNA maturation [118]; however, HNRNPA2B1 can inhibit miR-222, miR-29b-3p, and miR-29a-3p expression and affect the levels of m6A-modified miRNAs, leading to endocrine resistance in breast cancer cells [129]. Additionally, inhibition of miRNA processing by m6A modified may be associated with the recognition of methylation-modified miRNA degradation proteins [130]. In conclusion, the regulation of m6A-modified miRNAs has an important impact on cancer development. However, the regulatory mechanisms of m6A-modified repressive miRNAs need to be further explored.

### lncRNAs

lncRNAs represent a category of non-coding RNAs that have a length exceeding 200 nucleotides. Similar to mRNAs, most lncRNAs have five caps, can be spliced and polyadenylated, and can influence gene expression and cellular biology, particularly in cancer development [30, 131, 132].

### m6A regulates lncRNA structure

m6A methylation can also act as a structural switch in lncRNAs. Local changes in the m6A site of metastasis-associated lung adenocarcinoma transcript 1 (MALAT1) induce structural changes that increase the accessibility of the U5 channel for recognition and binding by HNRNPC [136].

### m6A regulates lncRNA stability

m6A can promote multiple tumor progressions by increasing lncRNA stability [134–136]. The competitive endogenous RNA (ceRNA) hypothesis refers to coding or non-coding RNAs containing miRNA response elements that compete for binding miRNA sites, thereby inhibiting miRNA activity [137]. m6A modification has been shown to regulate lncRNA stability by mediating ceRNA models, like FAM225A, an lncRNA that significantly connects to the proliferative, migrative, and invasion capacities of nasopharyngeal carcinoma (NPC) cells. Mechanistically, m6A modification enhances FAM225A stability, and FAM225A acts as a ceRNA to amplify miR⁃590⁃3p and miR1275, leading to integrin β3 (ITGB3) upregulation and FAK/PI3K/Akt signaling activation, thereby promoting the growth and metastasis of NPC cells [138]. The modification of m6A could improve the stability of RHPN1⁃AS1 methylated transcripts by reducing RNA degradation, leading to RHPN1⁃AS1 upregulation in epithelial ovarian cancer; RHPN1⁃AS1 increases LETM1 expression and activates the FAK/PI3K/Akt signaling pathway through spongy miR-596, leading to cancer cell proliferation and metastasis [139]. In HCC, the lncRNA LINC00958 increases hepatoma-derived growth factor (HDGF) expression by sponging miR-3619-5p, and METTL3 can mediate the positive regulation of the LINC00958/miR-3619-5p/HDGF axis through m6A modification to promote HCC progression [140]. Additionally, m6A may directly affected the binding of miRNAs to lncRNAs. Yang et al. showed that lncRNA 1281 modulates ESC differentiation by associating with the let-7 family of miRNAs. Moreover, several modification sites of m6A were observed in lncRNA 1281 and these are critical for the binding of let-7 [141].

### m6A promotes XIST-mediated gene silencing

The lncRNA X-inactive specific transcript (XIST) mediates the silencing of X chromosome-located genes at the transcriptional level. Through the formation of METTL3-WTAP-RBM15 polymers, XIST can regulate gene silencing at the transcriptional level [142], and decreasing the level of m6A modification affects the ability of XIST to function. Furthermore, YTHDC1 recognizes m6A residues in XIST and mediates its subsequent transcriptional silencing [143].

### circRNAs

circRNAs are ncRNAs that lack 3′ and 5′ terminals, are commonly derived from pre-mRNA by variable shear processing, and are characterized by tissue-specific expression, sequence conservation, and structural stability [144].

### m6A regulates the biogenesis of circRNAs

CircRNA biogenesis differs from canonical splicing in four splicing mechanisms: intron cyclization, exon skipping (Lariat-driven circularization), and direct anti-splicing (RBP-driven circularization and base-pairing-driven circularization) [145]. The generation of circRNAs with open reading frames in male germ cells is dramatically enhanced by m6A, whereas reverse splicing of circRNAs occurs mainly at m6A-rich sites. m6A and circRNA abundance increased significantly after knocking down ALKBH5 in germ cells, whereas knockdown of METTL3 had the opposite effect [146]. In addition, Di Timoteo et al. analyzed the mechanism of m6A modification in the production and translational regulation of circZNF609 and found that m6A regulates circRNA biogenesis in a YTHDC1/METTL3-dependent manner [147]. Similarly, in colorectal cancer, the modifications of m6A can be installed by METTL3 in the reverse complementary sequence of circ1662 flanking introns and promote circ1662 production according to an intron pairing-driven cyclization pattern, leading to invasive metastasis in colorectal cancer [148].

### m6A affects the cytoplasmic export of circRNAs

CircRNAs are biosynthesized in the nucleus and either retained in the nucleus or exported to the cytoplasm. CircNSUN2 is an important oncogenic cyclic RNA, and Chen et al. found that m6A modifications can be recognized by YTHDC1 and facilitate the output of circNSUN2. By generating the RNA–protein ternary complexes of circNSUN2/IGF2BP2/HMGA2 and enhancing HMGA2 mRNA stability, circNSUN2 could facilitate the metastasis of colorectal cancer to the liver [149]. The promotion of m6A-induced cytoplasmic export of circGFRα1 through the GGACU sequence has been attributed to METTL14 [150].

### m6A methylation mediates the degradation of circRNA

Compared with their parent linear RNA, circRNAs have a more stable circular structure and are less susceptible to degradation by nucleic acid exonucleases [151]. However, the mechanisms underlying circRNA degradation remain unclear. It is now known that m6A is capable of mediating circRNA degradation via the intranuclear cleavage pathway. HRSP12 heat-responsive protein 12 (HRSP12) is a splice protein that links YTHDF2 and RNaseP/MRP (nucleic acid endonuclease) to form the complex of YTHDF2-HRSP12-RNaseP/MRP, with YTHDF2 acting as a guide factor; RNaseP/MRP performs endonuclease functions when the m6A-modified circRNA is identified by YTHDF2. The involvement of HRSP12 greatly increases the effectiveness of intranuclear lysis cleavage, followed by selected downregulation of m6A-modified circRNAs, resulting in the functional changes of the target genes regulated by circRNA [152].

### Modification of m6A modulates the ceRNA machinery of circRNA

CircRNAs usually have multiple miRNA adsorption sites that bind miRNAs and inhibit their functions, whereas m6A-modified circRNAs enhance miRNA functions by regulating the binding of circRNAs to microRNAs [153]. In kidney renal clear cell carcinoma (KIRC), circRNAs act as miRNA ‘sponges' to regulate METTL14 mRNA expression, thereby influencing the oncogenic role of METTL14 in KIRC [154]. Chen et al. demonstrated that the modification of m6A could stabilize circ0000069, which could sponge miR-4426, and thus promote the proliferation and migration of cervical cancer cells [155]. Similarly, the ceRNA activity of circMETTL3 allows it to sequester miR-31-5p, resulting in the upregulation of cyclin-dependent kinases (CDK1), thereby promoting breast cancer progression [156]. In parallel to the direct modification of circRNA, m6A can influence the functionality of circRNA by altering the methylation levels of downstream molecules. For example, circ_104075 promotes hepatocarcinogenesis through YAP binding to miR-582-3p, while YAP m6A modification could promote the binding between YAP and miR-382-5P, leading to the suppression of YAP and consequently, the promotion of hepatocarcinogenesis by circ_104075 [157]. Therefore, m6A may be important for interactions between circRNAs and miRNAs.

### m6A regulates the translation of circRNAs

Although circRNAs lack a poly A tail, they cannot perform translational functions in a cap-dependent manner [158]. However, some circRNAs can translate peptides in a non-cap-dependent manner, such as through the internal ribosome entry sites (IRES) [151] and m6A pathway [159]. m6A-driven circRNA translation is enhanced by eukaryotic initiation factor 4 gamma 2 (eIF4G2) and METTL3/14, initiated by YTHDF3, and inhibited by FTO [147, 158]. In addition, circRNAs containing m6A motifs are involved in the IRES-driven translation pathway, and translation efficiency is regulated by the level of m6A [137]. For example, in gliomas, circFBXW7 can translate into a new 21-kDa protein by driving IRES under the influence of m6A, further demonstrating that m6A methylation modifications can affect circRNA coding [160]. Legnini et al. found that circZNF609 is highly methylated by m6A-Seq analysis; these findings imply a potential connection between these two modes of translation that does not rely on a cap structure [151, 161]. It has been demonstrated that the hepatitis B virus X protein is capable of enhancing the expression of METTL3 and promoting m6A modification through circARL3, thus promoting the reverse shear and translation of circARL3 [162]. In cervical cancer, the m6A-modified circE7 is translated into an E7 protein that regulates cervical cancer cell proliferation [163]. The translational function of circRNAs enriches the translation machinery of the human genome; however, their specific regulatory mechanisms and potential biological functions remain to be explored.

In addition to the common ncRNAs mentioned above, m6A modifications have been identified in small nuclear RNAs (snRNAs) and ribosomal RNAs (rRNAs). It is now known that the m6A4220 amendment in 28S rRNA and m6A1832 amendment in 18S rRNA also play key roles in maintaining ribosomal translation dynamics [164, 165]. m6A modifications may affect splicing of U2 snRNA and U6 snRNA-specific precursor mRNA transcripts [44, 166].

### ncRNAs affect m6A modifications

As mentioned above, m6A affects tumor progression by regulating the production, shearing, cytoplasmic transport, translation, and degradation of ncRNAs. Notably, the aberrant expression of ncRNA can impact on the level of m6A modifications. For instance, miRNAs could be engaged in reprogramming efficiency and osteogenic lineage differentiation of mouse embryonic fibroblasts by targeting m6A regulatory proteins [167], and differentiation of osteogenic lineage [133] by targeting m6A regulatory proteins. By focusing on the 3′ UTR binding of mRNA, miR⁃33a could suppress the survival, metastasis, and growth of NSCLC cells, resulting in a decrease in METTL3 expression [168]. As a miR-186 target, METTL3 could promote hepatoblastoma progression [169]. In addition, miRNAs significantly affect protein expression. Abnormally high expression of YTHDF1 is also correlated with poor survival in patients with glioma, and research has shown that YTHDF1 accelerates glioma growth, while microRNA has-mir-3436 binds to the 3′ UTR region of YTHDF1 and negatively regulates YTHDF1 [151]. IGF2BP1 can be targeted by both miR-506 and miR-873. Upregulation of miR-873 and miR-506 reduces IGF2BP1 expression, destabilizes IGF2BP1 mRNA on its target genes *CD44, PTEN, MK167*, and *c-MYC*, and inhibits the proliferative and invasive capacities of GBM cells [170, 171]. Du et al. found that YTHDF2 is negatively regulated by miR-459 and inhibits the expression of MOB3B by recognizing the m6A site of Mps one binder kinase activator 3B (MOB3B) mRNA and inducing mRNA degradation, leading to the proliferation, migration, and invasion of PCa cells [172]. In an anti-osteosarcoma treatment study, miR-451a could stabilize the transcript of phosphoinositide-dependent protein kinase 1 (PDPK1) through the modification of m6A mediated by YTHDC1, and inhibit the signaling pathway associated with protein kinase B (PKB)/mammalian target of rapamycin (mTOR), thereby suppressing the progression of osteosarcoma [173]. In addition, the modification of m6A is also regulated by LncRNAs in cancer cells. For example, by stabilizing the transcripts of ARHGAP5 and promoting ARHGAP5 expression by recruiting METTL3, the chemoresistance of gastric cancer cells can be enhanced by lncRNA ARHGAP5-AS1 [174]. Similarly, Wang et al. found that the lncRNA NRON reduced the degradation of NANOG mRNA through ALKBH5 and enhanced the stability of NANOG, thereby promoting gastric cancer cells growth [175]. The lncRNA DMDRMR binds to IGF2BP3 and enhances IGF2BP3 activity in an m6A-dependent manner, thereby stabilizing FN1, LAMA5, COL6A1, and CDK4 expression, and promoting the transition of G1/S in KIRC [176]. In addition, lncRNA OIP5-AS1 increases IGF2BP2 expression by inhibiting miR-129-5p, which promotes glioma cell proliferation, inhibits apoptosis, and enhances chemoresistance to TMZ [177]. In addition, circRNA PTPRA could suppress BCa development and progression of downstream mRNAs, which are modified by m6A, leading to a decrease in the mRNA stability of the oncogenes *MYC* and *FSCN1* [178]. In conclusion, ncRNAs play an important role in the regulation of m6A modifications, and the associated regulatory mechanisms are instrumental in the progression of various tumors. This finding not only deepens our knowledge of the role of ncRNAs in regulating tumor disease onset and progression, but also suggests a novel avenue for investigating the mechanisms underlying the regulation of gene expression in cancer.

### Potential clinical applications of m6A in cancer

**Fig. 4:**
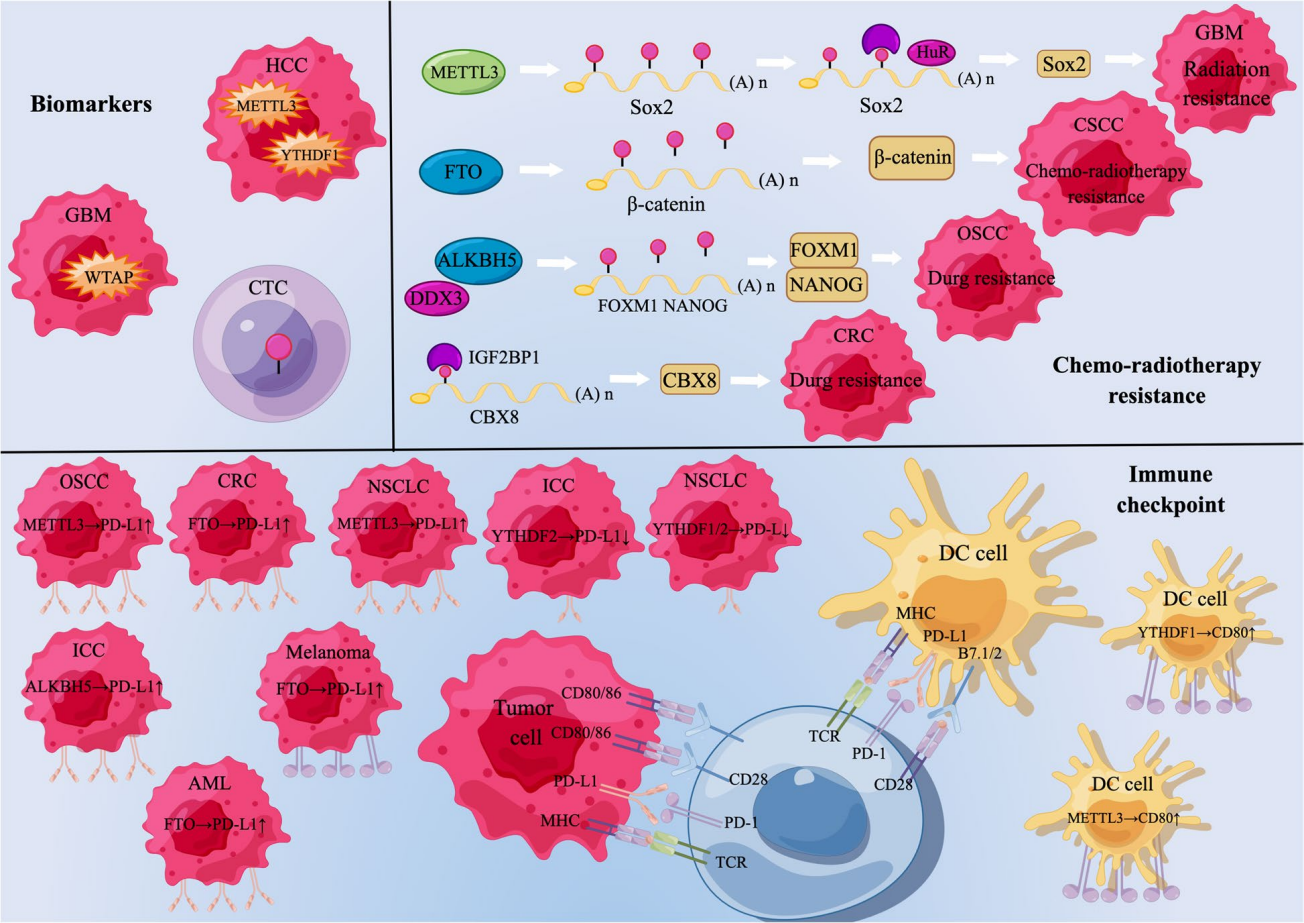
Potential clinical value of m6A modification. m6A-modified regulators may serve as potential markers for tumor diagnosis and prognosis and play an important role in radiotherapy resistance and immune checkpoint therapy in tumors

Based on these findings, it is evident that m6A modification is closely associated with tumor development. Several researchers have concentrated on the clinical application of m6A modification (Fig. 4).

### m6A as a tumor biomarker

Based on the analysis using The Cancer Genome Atlas, differential expression of the proteins associated with m6A in variety of tumors have been discovered. Additionally, high levels of YTHDF1 and METTL3 are indicative of a poor prognosis in patients suffering from HCC [90, 179]. Similarly, high expression of WTAP has been linked to worsened prognosis in glioma patients [180]. In addition, it has been shown that m6A levels are significantly higher in circulating tumor cells (CTC) than in whole blood in lung cancer patients, suggesting that testing m6A levels of CTC may be a potential non-invasive method of diagnosing cancer [109]. Thus, m6A modulator expression has the potential to diagnose tumor occurrence and predict the outcome of tumor patients as a biomarker.

### m6A is involved in chemo-radiotherapy resistance of tumors

m6A modification has a profound consequences for tumors, leading to new ideas for their treatment. m6A dysregulation is associated with resistance to radiotherapy and chemotherapy in tumor cells. In gliomas, METTL3-mediated m6A modification enhances SOX2 stability and DNA repair, thereby enhancing the radiation resistance of CSCs [181]. In addition, METTL3 was upregulated in drug-resistant osteosarcoma and nasopharyngeal carcinoma cell lines [182]. In cervical cancer, FTO regulates catenin expression by reducing m6A levels in mRNA transcripts, thereby enhancing resistance to chemo-radiotherapy [183]. The knockdown of the FTO gene has a significant sensitizing effect on tyrosine kinase inhibitor treatment-resistant leukemia cells [184]. Human RNA helicase DDX3 is involved in chemoresistance in oral squamous cell carcinoma (OSCC) by directly regulating ALKBH5, leading to reduced m6A methylation of FOXM1 and NANOG transcripts [185]. IGF2BP1 specifically combines with the mature mRNA of CBX8 and promotes CBX8 production depending on the presence of m6A modifications, thereby promoting colorectal cancer growth and reducing chemotherapy sensitivity [186]. These findings emphasize the therapeutic potential of directed m6A modulators in tumors resistant to drug therapy.

### m6A and tumor immunotherapy

m6A modifications are critical in the innate and adaptive immune response, indicating a potentially impactful effect of m6A modifications on tumor immunology [187]. T cells are central to the regulation of the adaptive immune response, and studies have demonstrated that the modification of m6A is significantly implicated in the differentiation and homeostasis of T cells [22]. Dendritic cells (DCs) perform key roles in antigen presentation in the innate and acquired immune system [188]. Han et al. found that YTHDF1 deficiency results in increased NK and CD8 + T cells infiltration in melanoma. This is because YTHDF1 knockdown restricts m6A-modified lysosomal histone protease translation in DCs, leading to delayed neoantigen degradation, thereby promoting the cross-presentation of antigens, enhancing the cross-triggering of CD8 + T cells, and enhancing anti-PD-L1 therapeutic efficacy [189]. In addition, in DCs Triap, CD80, and CD40 expression is also influenced by the methylation of m6A mediated by METTL3, thereby promoting T cell responses and cytokine production [190]. Monoclonal antibody blockade therapies targeting immune checkpoints are a new hot topic in cancer treatment [191]. m6A modifications also play essential roles in regulating immune checkpoints. In OSCC, METTL3-induced m6A modification enhances PD-L1 expression and suppresses CD8 + T cell activation, leading to tumor progression [192]. Similarly, METTL3 mediates the m6A-modified circIGF2BP3 to stabilize *OTUB1* mRNA by upregulating Plakophilin 3 (PKP3) expression, thereby suppressing the immune response in NSCLC [193]. Yang et al. showed that FTO knockout sensitized melanoma cells to IFN-γ and sensitized melanomas to anti-PD-1 treatment in mice [194]. In colon cancer, FTO depletion reduces PD-L1 expression independently of IFN-γ signaling [195]. FTO knockdown also results in the significant suppression of PD-L2 and PD-L1 expression in AML [196]. In addition, Liu et al. showed that FTO-mediated demethylation of m6A enables melanoma cells to evade immune surveillance and suppress T-cell immune responses in tumors by regulating glycolytic processing [197]. ALKBH5 modulates the sensitivity of the anti-PD-1 therapeutic response by regulating m6A abundance and RNA stability of Mct4/slc16a3 in the tumor microenvironment [65]. Furthermore, in intrahepatic cholangiocarcinoma, the knockdown of ALKBH5 increases m6A abundance and promotes PD-L1 mRNA degradation in a YTHDF2-dependent manner [198]. Another study showed that depletion of YTHDF2 and YTHDF1 potentiates the expression of PD-L1 in NSCLC cells [199]. Therefore, research based on the combination of m6A and tumor immunotherapy holds significant potential in the therapy of tumors and could present new treatment options for cancer patients.

## Conclusions

In summary, m6A has a major impact on tumor proliferation, apoptosis, migration, invasion, energy processing, and tolerance to radiotherapy. Elucidating the molecular machinery of m6A in RNA and its impact on tumor behavior will provide an essential basis for tumor diagnosis, treatment and prognosis. Research on m6A modification in tumors is still at an early stage, and the identified m6A modification-related proteins may only be a small fraction of the total; the unknown regulators still need to be further explored and identified. Dysregulation of m6A levels and its modifying proteins appears to be a "double-edged sword" in suppressing and promoting cancer, and rational interpretation of the controversial findings remains challenging, including, for example, the relative paucity of inter-regulatory mechanisms between m6A modifications and ncRNAs. Combination therapy targeting m6A and immune checkpoints in tumors holds great promise and deserves further investigation. In addition, the mechanism by which m6A, an important RNA epigenetic modification, can synergistically control gene expression with other epigenetic modifications, such as DNA and histones, needs to be further explored for viable application in clinical practice.

## Data Availability

Data sharing is not applicable to this article as no datasets were generated or analyzed during the current study.

## Abbreviations

ALKBH3/5: AlkB homolog 3/5 RNA demethylase
AML: Acute myeloid leukemia
CBLL1: CBL proto-oncogene-like 1
ceRNA: Competitive endogenous RNA
circRNA: Circular RNA
CRC: Colorectal cancer
CTD: Carboxyl-terminal domain
DC: Dendritic cell
EIF3: Eukaryotic initiation factor 3
FMRP: Fragile X mental retardation protein
FTO: Fat mass and obesity-associated protein
HCC: Hepatocellular carcinoma
HDGF: Hepatoma-derived growth factor
HNRN: Heteronuclear RNA protein
HRSP12: Heat-responsive protein 12
IGF2BP: Insulin-like growth factor 2 mRNA-binding protein
IRES: Internal ribosome entry site
KDM3B: Lysine demethylase 3B
KIRC: Kidney renal clear cell carcinoma
lncRNA: Long non-coding RNA
LRPPRC: Leucine-rich pentatricopeptide repeat containing protein
m6A: N6-methyladenosine
METTL: Methyltransferase-like
miRNA: MicroRNA
ncRNA: Non-coding RNA
NSCLC: Non-small cell lung cancer
PCIF1: Phosphorylated CTD-interacting factor 1
PD-L1: Programmed death ligand 1
PRRC2A: Proline-rich coiled-coil 2 A
PTEN: Phosphate and tension homology deleted on chromosome 10
RBM: RNA-binding motif protein
RBP: RNA-binding protein
SND1: Secondary wall-associated NAC domain protein 1
snRNA: Small nuclear RNA
SRSF: Serine/arginine-rich splicing factor
UTR: Untranslated region
VIRMA: Vir-like m6A methyltransferase-associated protein
WTAP: WT1-associated protein and RNA-binding motif protein
XIST: X-inactive specific transcript
ZC3H13: Zinc-finger CCCH-type containing 13
BCa: Bladder cancer

## References

[1] Mi S, Shi Y, Dari G, Yu Y (2022). Function of m6A and its regulation of domesticated animals’ complex traits. J Anim Sci.

[2] Boccaletto P, Stefaniak F, Ray A, Cappannini A, Mukherjee S, Purta E (2022). MODOMICS: a database of RNA modification pathways. 2021 update. Nucleic Acids Res.

[3] Nie WF (2021). DNA methylation: from model plants to vegetable crops. Biochem Soc Trans.

[4] An Y, Duan H (2022). The role of m6A RNA methylation in cancer metabolism. Mol Cancer.

[5] Shvedunova M, Akhtar A (2022). Modulation of cellular processes by histone and non-histone protein acetylation. Nat Rev Mol Cell Biol.

[6] Pacheco MB, Camilo V, Henrique R, Jerónimo C (2022). Epigenetic editing in prostate cancer: challenges and opportunities. Epigenetics.

[7] D D, S MM, S S, M SD, L U, S O, et al. Topology of the human and mouse m6A RNA methylomes revealed by m6A-seq. Nature. 2012;485(7397). Available from: https://pubmed.ncbi.nlm.nih.gov/22575960/. [Cited 2023 Mar 26]. 10.1038/nature11112 22575960

[8] Li X, Xiong X, Wang K, Wang L, Shu X, Ma S (2016). Transcriptome-wide mapping reveals reversible and dynamic N(1)-methyladenosine methylome. Nat Chem Biol.

[9] Dominissini D, Nachtergaele S, Moshitch-Moshkovitz S, Peer E, Kol N, Ben-Haim MS (2016). The dynamic N(1)-methyladenosine methylome in eukaryotic messenger RNA. Nature.

[10] Edelheit S, Schwartz S, Mumbach MR, Wurtzel O, Sorek R (2013). Transcriptome-wide mapping of 5-methylcytidine RNA modifications in bacteria, archaea, and yeast reveals m5C within archaeal mRNAs. PLoS Genet.

[11] Roundtree IA, Evans ME, Pan T, He C (2017). Dynamic RNA modifications in gene expression regulation. Cell.

[12] Bodi Z, Bottley A, Archer N, May ST, Fray RG (2015). Yeast m6A Methylated mRNAs are enriched on translating ribosomes during meiosis, and under rapamycin treatment. PLoS One.

[13] Csepany T, Lin A, Baldick CJ, Beemon K (1990). Sequence specificity of mRNA N6-adenosine methyltransferase. J Biol Chem.

[14] Narayan P, Rottman FM (1988). An in vitro system for accurate methylation of internal adenosine residues in messenger RNA. Science.

[15] Linder B, Grozhik AV, Olarerin-George AO, Meydan C, Mason CE, Jaffrey SR (2015). Single-nucleotide-resolution mapping of m6A and m6Am throughout the transcriptome. Nat Methods.

[16] Huang J, Yin P (2018). Structural Insights into N6-methyladenosine (m6A) Modification in the Transcriptome. Genomics Proteomics Bioinformatics.

[17] Dai D, Wang H, Zhu L, Jin H, Wang X (2018). N6-methyladenosine links RNA metabolism to cancer progression. Cell Death Dis.

[18] Fustin JM, Doi M, Yamaguchi Y, Hida H, Nishimura S, Yoshida M (2013). RNA-methylation-dependent RNA processing controls the speed of the circadian clock. Cell.

[19] Lin Z, Hsu PJ, Xing X, Fang J, Lu Z, Zou Q (2017). Mettl3-/Mettl14-mediated mRNA N6-methyladenosine modulates murine spermatogenesis. Cell Res.

[20] Mendel M, Chen KM, Homolka D, Gos P, Pandey RR, McCarthy AA (2018). Methylation of Structured RNA by the m6A Writer METTL16 Is Essential for Mouse Embryonic Development. Mol Cell.

[21] Aguilo F, Zhang F, Sancho A, Fidalgo M, Di Cecilia S, Vashisht A (2015). Coordination of m(6)A mRNA Methylation and Gene Transcription by ZFP217 Regulates Pluripotency and Reprogramming. Cell Stem Cell.

[22] Li HB, Tong J, Zhu S, Batista PJ, Duffy EE, Zhao J (2017). m6A mRNA methylation controls T cell homeostasis by targeting the IL-7/STAT5/SOCS pathways. Nature.

[23] Zhou J, Wan J, Gao X, Zhang X, Jaffrey SR, Qian SB (2015). Dynamic m(6)A mRNA methylation directs translational control of heat shock response. Nature.

[24] Mathiyalagan P, Adamiak M, Mayourian J, Sassi Y, Liang Y, Agarwal N (2019). FTO-Dependent N6-Methyladenosine regulates cardiac function during remodeling and repair. Circulation.

[25] Ben-Haim MS, Moshitch-Moshkovitz S, Rechavi G (2015). FTO: linking m6A demethylation to adipogenesis. Cell Res.

[26] Shen F, Huang W, Huang JT, Xiong J, Yang Y, Wu K (2015). Decreased N(6)-methyladenosine in peripheral blood RNA from diabetic patients is associated with FTO expression rather than ALKBH5. J Clin Endocrinol Metab.

[27] Zheng G, Dahl JA, Niu Y, Fedorcsak P, Huang CM, Li CJ (2013). ALKBH5 is a mammalian RNA demethylase that impacts RNA metabolism and mouse fertility. Mol Cell.

[28] Angelova MT, Dimitrova DG, Dinges N, Lence T, Worpenberg L, Carré C (2018). The Emerging Field of Epitranscriptomics in Neurodevelopmental and Neuronal Disorders. Front Bioeng Biotechnol.

[29] Zhao J, Lu L (2021). Interplay between RNA Methylation Eraser FTO and Writer METTL3 in Renal Clear Cell Carcinoma Patient Survival. Recent Pat Anticancer Drug Discov.

[30] Yang X, Zhang S, He C, Xue P, Zhang L, He Z (2020). METTL14 suppresses proliferation and metastasis of colorectal cancer by down-regulating oncogenic long non-coding RNA XIST. Mol Cancer.

[31] Niu Y, Zhao X, Wu YS, Li MM, Wang XJ, Yang YG (2013). N6-methyl-adenosine (m6A) in RNA: an old modification with a novel epigenetic function. Genomics Proteomics Bioinformatics.

[32] Peng F, Xu J, Cui B, Liang Q, Zeng S, He B (2021). Oncogenic AURKA-enhanced N6-methyladenosine modification increases DROSHA mRNA stability to transactivate STC1 in breast cancer stem-like cells. Cell Res.

[33] Li B, Jiang J, Assaraf YG, Xiao H, Chen ZS, Huang C (2020). Surmounting cancer drug resistance: New insights from the perspective of N6-methyladenosine RNA modification. Drug Resist Updat.

[34] Yi YC, Chen XY, Zhang J, Zhu JS (2020). Novel insights into the interplay between m6A modification and noncoding RNAs in cancer. Mol Cancer.

[35] Deng X, Qing Y, Horne D, Huang H, Chen J (2023). The roles and implications of RNA m6A modification in cancer. Nat Rev Clin Oncol.

[36] Jia G, Fu Y, Zhao X, Dai Q, Zheng G, Yang Y (2011). N6-methyladenosine in nuclear RNA is a major substrate of the obesity-associated FTO. Nat Chem Biol.

[37] Liu J, Yue Y, Han D, Wang X, Fu Y, Zhang L (2014). A METTL3-METTL14 complex mediates mammalian nuclear RNA N6-adenosine methylation. Nat Chem Biol.

[38] Bokar JA, Shambaugh ME, Polayes D, Matera AG, Rottman FM (1997). Purification and cDNA cloning of the AdoMet-binding subunit of the human mRNA (N6-adenosine)-methyltransferase. RNA.

[39] Ping XL, Sun BF, Wang L, Xiao W, Yang X, Wang WJ (2014). Mammalian WTAP is a regulatory subunit of the RNA N6-methyladenosine methyltransferase. Cell Res.

[40] Liu ZX, Li LM, Sun HL, Liu SM (2018). Link Between m6A Modification and Cancers. Front Bioeng Biotechnol.

[41] Yue Y, Liu J, Cui X, Cao J, Luo G, Zhang Z (2018). VIRMA mediates preferential m6A mRNA methylation in 3’UTR and near stop codon and associates with alternative polyadenylation. Cell Discov.

[42] Wen J, Lv R, Ma H, Shen H, He C, Wang J (2018). Zc3h13 Regulates Nuclear RNA m6A Methylation and Mouse Embryonic Stem Cell Self-Renewal. Mol Cell.

[43] Warda AS, Kretschmer J, Hackert P, Lenz C, Urlaub H, Höbartner C (2017). Human METTL16 is a N6-methyladenosine (m6A) methyltransferase that targets pre-mRNAs and various non-coding RNAs. EMBO Rep.

[44] Pendleton KE, Chen B, Liu K, Hunter OV, Xie Y, Tu BP (2017). The U6 snRNA m6A Methyltransferase METTL16 Regulates SAM Synthetase Intron Retention. Cell.

[45] Lei K, Lin S, Yuan Q (2023). N6-methyladenosine (m6A) modification of ribosomal RNAs (rRNAs): Critical roles in mRNA translation and diseases. Genes Dis.

[46] Sendinc E, Valle-Garcia D, Dhall A, Chen H, Henriques T, Navarrete-Perea J (2019). PCIF1 Catalyzes m6Am mRNA Methylation to Regulate Gene Expression. Mol Cell.

[47] Chen H, Gu L, Orellana EA, Wang Y, Guo J, Liu Q (2020). METTL4 is an snRNA m6Am methyltransferase that regulates RNA splicing. Cell Res.

[48] Wang T, Kong S, Tao M, Ju S (2020). The potential role of RNA N6-methyladenosine in Cancer progression. Mol Cancer.

[49] Fedeles BI, Singh V, Delaney JC, Li D, Essigmann JM (2015). The AlkB Family of Fe(II)/α-Ketoglutarate-dependent Dioxygenases: Repairing Nucleic Acid Alkylation Damage and Beyond. J Biol Chem.

[50] Huang H, Weng H, Sun W, Qin X, Shi H, Wu H (2018). Recognition of RNA N6-methyladenosine by IGF2BP proteins enhances mRNA stability and translation. Nat Cell Biol.

[51] Wu R, Li A, Sun B, Sun JG, Zhang J, Zhang T (2019). A novel m6A reader Prrc2a controls oligodendroglial specification and myelination. Cell Res.

[52] Ruzzenente B, Metodiev MD, Wredenberg A, Bratic A, Park CB, Cámara Y (2012). LRPPRC is necessary for polyadenylation and coordination of translation of mitochondrial mRNAs. EMBO J.

[53] Kim GW, Imam H, Siddiqui A (2021). The RNA Binding Proteins YTHDC1 and FMRP Regulate the Nuclear Export of N6-Methyladenosine-Modified Hepatitis B Virus Transcripts and Affect the Viral Life Cycle. J Virol.

[54] Baquero-Perez B, Antanaviciute A, Yonchev ID, Carr IM, Wilson SA, Whitehouse A (2019). The Tudor SND1 protein is an m6A RNA reader essential for replication of Kaposi’s sarcoma-associated herpesvirus. Elife.

[55] Liu N, Dai Q, Zheng G, He C, Parisien M, Pan T (2015). N(6)-methyladenosine-dependent RNA structural switches regulate RNA-protein interactions. Nature.

[56] Li Y, Xia L, Tan K, Ye X, Zuo Z, Li M (2020). N6-Methyladenosine co-transcriptionally directs the demethylation of histone H3K9me2. Nat Genet.

[57] Liu J, Dou X, Chen C, Chen C, Liu C, Xu MM (2020). N 6-methyladenosine of chromosome-associated regulatory RNA regulates chromatin state and transcription. Science.

[58] Yang X, Liu QL, Xu W, Zhang YC, Yang Y, Ju LF (2019). m6A promotes R-loop formation to facilitate transcription termination. Cell Res.

[59] Ke S, Alemu EA, Mertens C, Gantman EC, Fak JJ, Mele A (2015). A majority of m6A residues are in the last exons, allowing the potential for 3’ UTR regulation. Genes Dev.

[60] Molinie B, Wang J, Lim KS, Hillebrand R, Lu ZX, Van Wittenberghe N (2016). m(6)A-LAIC-seq reveals the census and complexity of the m(6)A epitranscriptome. Nat Methods.

[61] Tian B, Manley JL (2017). Alternative polyadenylation of mRNA precursors. Nat Rev Mol Cell Biol.

[62] Yuan F, Hankey W, Wagner EJ, Li W, Wang Q (2021). Alternative polyadenylation of mRNA and its role in cancer. Genes Dis.

[63] Zhang Y, Liu L, Qiu Q, Zhou Q, Ding J, Lu Y (2021). Alternative polyadenylation: methods, mechanism, function, and role in cancer. J Exp Clin Cancer Res.

[64] Zhang T, Zhang SW, Zhang SY, Gao SJ, Chen Y, Huang Y (2021). m6A-express: uncovering complex and condition-specific m6A regulation of gene expression. Nucleic Acids Res.

[65] Li N, Kang Y, Wang L, Huff S, Tang R, Hui H (2020). ALKBH5 regulates anti-PD-1 therapy response by modulating lactate and suppressive immune cell accumulation in tumor microenvironment. Proc Natl Acad Sci U S A.

[66] Kasowitz SD, Ma J, Anderson SJ, Leu NA, Xu Y, Gregory BD (2018). Nuclear m6A reader YTHDC1 regulates alternative polyadenylation and splicing during mouse oocyte development. PLoS Genet.

[67] Zhou KI, Shi H, Lyu R, Wylder AC, Matuszek Ż, Pan JN (2019). Regulation of Co-transcriptional Pre-mRNA Splicing by m6A through the Low-Complexity Protein hnRNPG. Mol Cell.

[68] Huang XT, Li JH, Zhu XX, Huang CS, Gao ZX, Xu QC (2021). HNRNPC impedes m6A-dependent anti-metastatic alternative splicing events in pancreatic ductal adenocarcinoma. Cancer Lett.

[69] Edupuganti RR, Geiger S, Lindeboom RGH, Shi H, Hsu PJ, Lu Z (2017). N6-methyladenosine (m6A) recruits and repels proteins to regulate mRNA homeostasis. Nat Struct Mol Biol.

[70] Liu N, Zhou KI, Parisien M, Dai Q, Diatchenko L, Pan T (2017). N6-methyladenosine alters RNA structure to regulate binding of a low-complexity protein. Nucleic Acids Res.

[71] Camper SA, Albers RJ, Coward JK, Rottman FM (1984). Effect of undermethylation on mRNA cytoplasmic appearance and half-life. Mol Cell Biol.

[72] Li D, Cai L, Meng R, Feng Z, Xu Q (2020). METTL3 Modulates Osteoclast Differentiation and Function by Controlling RNA Stability and Nuclear Export. Int J Mol Sci.

[73] Covelo-Molares H, Obrdlik A, Poštulková I, Dohnálková M, Gregorová P, Ganji R (2021). The comprehensive interactomes of human adenosine RNA methyltransferases and demethylases reveal distinct functional and regulatory features. Nucleic Acids Res.

[74] Roundtree IA, Luo GZ, Zhang Z, Wang X, Zhou T, Cui Y (2017). YTHDC1 mediates nuclear export of N6-methyladenosine methylated mRNAs. Elife.

[75] Dong S, Wu Y, Liu Y, Weng H, Huang H (2021). N6-methyladenosine Steers RNA Metabolism and Regulation in Cancer. Cancer Commun.

[76] Zaccara S, Ries RJ, Jaffrey SR (2019). Reading, writing and erasing mRNA methylation. Nat Rev Mol Cell Biol.

[77] Wang X, Lu Z, Gomez A, Hon GC, Yue Y, Han D (2014). N6-methyladenosine-dependent regulation of messenger RNA stability. Nature.

[78] Du H, Zhao Y, He J, Zhang Y, Xi H, Liu M (2016). YTHDF2 destabilizes m(6)A-containing RNA through direct recruitment of the CCR4-NOT deadenylase complex. Nat Commun.

[79] Shi H, Wang X, Lu Z, Zhao BS, Ma H, Hsu PJ (2017). YTHDF3 facilitates translation and decay of N6-methyladenosine-modified RNA. Cell Res.

[80] Chai RC, Chang YZ, Chang X, Pang B, An SY, Zhang KN (2021). YTHDF2 facilitates UBXN1 mRNA decay by recognizing METTL3-mediated m6A modification to activate NF-κB and promote the malignant progression of glioma. J Hematol Oncol.

[81] Fang R, Chen X, Zhang S, Shi H, Ye Y, Shi H (2021). EGFR/SRC/ERK-stabilized YTHDF2 promotes cholesterol dysregulation and invasive growth of glioblastoma. Nat Commun.

[82] Villa GR, Hulce JJ, Zanca C, Bi J, Ikegami S, Cahill GL (2016). An LXR-Cholesterol Axis Creates a Metabolic Co-Dependency for Brain Cancers. Cancer Cell.

[83] Wojtas MN, Pandey RR, Mendel M, Homolka D, Sachidanandam R, Pillai RS (2017). Regulation of m6A Transcripts by the 3’→5’ RNA Helicase YTHDC2 Is Essential for a Successful Meiotic Program in the Mammalian Germline. Mol Cell.

[84] Bailey AS, Batista PJ, Gold RS, Chen YG, de Rooij DG, Chang HY (2017). The conserved RNA helicase YTHDC2 regulates the transition from proliferation to differentiation in the germline. Elife.

[85] Jain D, Puno MR, Meydan C, Lailler N, Mason CE, Lima CD (2018). ketu mutant mice uncover an essential meiotic function for the ancient RNA helicase YTHDC2. Elife.

[86] Hou P, Meng S, Li M, Lin T, Chu S, Li Z (2021). LINC00460/DHX9/IGF2BP2 complex promotes colorectal cancer proliferation and metastasis by mediating HMGA1 mRNA stability depending on m6A modification. J Exp Clin Cancer Res.

[87] Hsu PJ, Zhu Y, Ma H, Guo Y, Shi X, Liu Y (2017). Ythdc2 is an N6-methyladenosine binding protein that regulates mammalian spermatogenesis. Cell Res.

[88] Li Z, Weng H, Su R, Weng X, Zuo Z, Li C (2017). FTO Plays an Oncogenic Role in Acute Myeloid Leukemia as a N6-Methyladenosine RNA Demethylase. Cancer Cell.

[89] Liu J, Ren D, Du Z, Wang H, Zhang H, Jin Y (2018). m6A demethylase FTO facilitates tumor progression in lung squamous cell carcinoma by regulating MZF1 expression. Biochem Biophys Res Commun.

[90] Chen M, Wei L, Law CT, Tsang FHC, Shen J, Cheng CLH (2018). RNA N6-methyladenosine methyltransferase-like 3 promotes liver cancer progression through YTHDF2-dependent posttranscriptional silencing of SOCS2. Hepatology.

[91] Yue B, Song C, Yang L, Cui R, Cheng X, Zhang Z (2019). METTL3-mediated N6-methyladenosine modification is critical for epithelial-mesenchymal transition and metastasis of gastric cancer. Mol Cancer.

[92] Vu LP, Pickering BF, Cheng Y, Zaccara S, Nguyen D, Minuesa G (2017). The N6-methyladenosine (m6A)-forming enzyme METTL3 controls myeloid differentiation of normal hematopoietic and leukemia cells. Nat Med.

[93] Hua W, Zhao Y, Jin X, Yu D, He J, Xie D (2018). METTL3 promotes ovarian carcinoma growth and invasion through the regulation of AXL translation and epithelial to mesenchymal transition. Gynecol Oncol.

[94] Chen H, Gao S, Liu W, Wong CC, Wu J, Wu J (2021). RNA N6-Methyladenosine Methyltransferase METTL3 Facilitates Colorectal Cancer by Activating the m6A-GLUT1-mTORC1 Axis and Is a Therapeutic Target. Gastroenterology.

[95] Choe J, Lin S, Zhang W, Liu Q, Wang L, Ramirez-Moya J (2018). mRNA circularization by METTL3-eIF3h enhances translation and promotes oncogenesis. Nature.

[96] Su R, Dong L, Li Y, Gao M, He PC, Liu W (2022). METTL16 exerts an m6A-independent function to facilitate translation and tumorigenesis. Nat Cell Biol.

[97] Liu Z, Chen Y, Wang L, Ji S (2021). ALKBH5 Promotes the Proliferation of Glioma Cells via Enhancing the mRNA Stability of G6PD. Neurochem Res.

[98] Wang X, Zhao BS, Roundtree IA, Lu Z, Han D, Ma H (2015). N(6)-methyladenosine Modulates Messenger RNA Translation Efficiency. Cell.

[99] Li A, Chen YS, Ping XL, Yang X, Xiao W, Yang Y (2017). Cytoplasmic m6A reader YTHDF3 promotes mRNA translation. Cell Res.

[100] Bai Y, Yang C, Wu R, Huang L, Song S, Li W (2019). YTHDF1 Regulates Tumorigenicity and Cancer Stem Cell-Like Activity in Human Colorectal Carcinoma. Front Oncol.

[101] Zhang C, Huang S, Zhuang H, Ruan S, Zhou Z, Huang K (2020). YTHDF2 promotes the liver cancer stem cell phenotype and cancer metastasis by regulating OCT4 expression via m6A RNA methylation. Oncogene.

[102] Meyer KD, Patil DP, Zhou J, Zinoviev A, Skabkin MA, Elemento O (2015). 5’ UTR m(6)A Promotes Cap-Independent Translation. Cell.

[103] Tanabe A, Tanikawa K, Tsunetomi M, Takai K, Ikeda H, Konno J (2016). RNA helicase YTHDC2 promotes cancer metastasis via the enhancement of the efficiency by which HIF-1α mRNA is translated. Cancer Lett.

[104] Guttman M, Rinn JL (2012). Modular regulatory principles of large non-coding RNAs. Nature.

[105] Shen S, Zhang R, Jiang Y, Li Y, Lin L, Liu Z (2021). Comprehensive analyses of m6A regulators and interactive coding and non-coding RNAs across 32 cancer types. Mol Cancer.

[106] Alarcón CR, Lee H, Goodarzi H, Halberg N, Tavazoie SF (2015). N6-methyladenosine marks primary microRNAs for processing. Nature.

[107] Haussmann IU, Bodi Z, Sanchez-Moran E, Mongan NP, Archer N, Fray RG (2016). m6A potentiates Sxl alternative pre-mRNA splicing for robust Drosophila sex determination. Nature.

[108] Li Y, Xiao J, Bai J, Tian Y, Qu Y, Chen X (2019). Molecular characterization and clinical relevance of m6A regulators across 33 cancer types. Mol Cancer.

[109] Huang W, Qi CB, Lv SW, Xie M, Feng YQ, Huang WH (2016). Determination of DNA and RNA Methylation in Circulating Tumor Cells by Mass Spectrometry. Anal Chem.

[110] Chen T, Hao YJ, Zhang Y, Li MM, Wang M, Han W (2015). m(6)A RNA methylation is regulated by microRNAs and promotes reprogramming to pluripotency. Cell Stem Cell.

[111] Integrative Analysis of NSCLC Identifies LINC01234 as an Oncogenic lncRNA that Interacts with HNRNPA2B1 and Regulates miR-106b Biogenesis. Molecular Therapy. 2020 Jun 3;28(6):1479–93. 10.1016/j.ymthe.2020.03.010 PMC7264428 32246902

[112] Iwakawa HO, Tomari Y (2015). The functions of MicroRNAs: mRNA decay and translational repression. Trends Cell Biol.

[113] Td S, H O, T Y, Jy L, Cz H, Hs Y, et al. miRNA-30e regulates abnormal differentiation of small intestinal epithelial cells in diabetic mice by downregulating Dll4 expression. Cell proliferation. 2016;49(1). Available from: https://pubmed.ncbi.nlm.nih.gov/26786283/. [Cited 2023 Apr 11]. 10.1111/cpr.12230 PMC6496571 26786283

[114] Treiber T, Treiber N, Meister G (2019). Regulation of microRNA biogenesis and its crosstalk with other cellular pathways. Nat Rev Mol Cell Biol.

[115] Kim B, Jeong K, Kim VN (2017). Genome-wide Mapping of DROSHA cleavage sites on primary MicroRNAs and Noncanonical Substrates. Mol Cell.

[116] Nguyen TA, Jo MH, Choi YG, Park J, Kwon SC, Hohng S (2015). Functional anatomy of the human microprocessor. Cell.

[117] Cr A, H L, H G, N H, Sf T. N6-methyladenosine marks primary microRNAs for processing. Nature. 2015;519(7544). Available from: https://pubmed.ncbi.nlm.nih.gov/25799998/. [Cited 2023 Mar 30]. 10.1038/nature14281 PMC4475635 25799998

[118] Alarcón CR, Goodarzi H, Lee H, Liu X, Tavazoie S, Tavazoie SF (2015). HNRNPA2B1 Is a Mediator of m(6)A-Dependent Nuclear RNA Processing Events. Cell.

[119] Han J, Wang JZ, Yang X, Yu H, Zhou R, Lu HC (2019). METTL3 promote tumor proliferation of bladder cancer by accelerating pri-miR221/222 maturation in m6A-dependent manner. Mol Cancer.

[120] Peng W, Li J, Chen R, Gu Q, Yang P, Qian W (2019). Upregulated METTL3 promotes metastasis of colorectal Cancer via miR-1246/SPRED2/MAPK signaling pathway. J Exp Clin Cancer Res.

[121] Ma JZ, Yang F, Zhou CC, Liu F, Yuan JH, Wang F (2017). METTL14 suppresses the metastatic potential of hepatocellular carcinoma by modulating N6 -methyladenosine-dependent primary MicroRNA processing. Hepatology.

[122] Shah A, Rashid F, Awan HM, Hu S, Wang X, Chen L (2017). The DEAD-Box RNA Helicase DDX3 Interacts with m6A RNA Demethylase ALKBH5. Stem Cells Int.

[123] Chen Z, Chen X, Lei T, Gu Y, Gu J, Huang J (2020). Integrative Analysis of NSCLC Identifies LINC01234 as an Oncogenic lncRNA that Interacts with HNRNPA2B1 and Regulates miR-106b Biogenesis. Mol Ther.

[124] Zhang J, Bai R, Li M, Ye H, Wu C, Wang C (2019). Excessive miR-25-3p maturation via N6-methyladenosine stimulated by cigarette smoke promotes pancreatic cancer progression. Nat Commun.

[125] Park MS, Araya-Secchi R, Brackbill JA, Phan HD, Kehling AC, Abd El-Wahab EW (2019). Multidomain Convergence of Argonaute during RISC assembly correlates with the formation of internal water clusters. Mol Cell.

[126] Liang XH, Nichols JG, Hsu CW, Vickers TA, Crooke ST (2019). mRNA levels can be reduced by antisense oligonucleotides via no-go decay pathway. Nucleic Acids Res.

[127] Becker WR, Ober-Reynolds B, Jouravleva K, Jolly SM, Zamore PD, Greenleaf WJ (2019). High-Throughput Analysis Reveals Rules for Target RNA Binding and Cleavage by AGO2. Mol Cell.

[128] Kim Y, Yeo J, Lee JH, Cho J, Seo D, Kim JS (2014). Deletion of human tarbp2 reveals cellular microRNA targets and cell-cycle function of TRBP. Cell Rep.

[129] Klinge CM, Piell KM, Tooley CS, Rouchka EC (2021). Author Correction: HNRNPA2/B1 is upregulated in endocrine-resistant LCC9 breast cancer cells and alters the miRNA transcriptome when overexpressed in MCF-7 cells. Sci Rep.

[130] Lee Y, Choe J, Park OH, Kim YK (2020). Molecular Mechanisms Driving mRNA Degradation by m6A Modification. Trends Genet.

[131] Kopp F, Mendell JT (2018). Functional Classification and Experimental Dissection of Long Noncoding RNAs. Cell.

[132] Chen J, Yu Y, Li H, Hu Q, Chen X, He Y (2019). Long non-coding RNA PVT1 promotes tumor progression by regulating the miR-143/HK2 axis in gallbladder cancer. Mol Cancer.

[133] Zhou KI, Parisien M, Dai Q, Liu N, Diatchenko L, Sachleben JR (2016). N(6)-Methyladenosine Modification in a Long Noncoding RNA Hairpin Predisposes Its Conformation to Protein Binding. J Mol Biol.

[134] Chang YZ, Chai RC, Pang B, Chang X, An SY, Zhang KN (2021). METTL3 enhances the stability of MALAT1 with the assistance of HuR via m6A modification and activates NF-κB to promote the malignant progression of IDH-wildtype glioma. Cancer Lett.

[135] Barros-Silva D, Lobo J, Guimarães-Teixeira C, Carneiro I, Oliveira J, Martens-Uzunova ES (2020). VIRMA-Dependent N6-Methyladenosine Modifications Regulate the Expression of Long Non-Coding RNAs CCAT1 and CCAT2 in Prostate Cancer. Cancers (Basel).

[136] Xue L, Li J, Lin Y, Liu D, Yang Q, Jian J (2021). m6 A transferase METTL3-induced lncRNA ABHD11-AS1 promotes the Warburg effect of non-small-cell lung cancer. J Cell Physiol.

[137] Carmeliet P, Jain RK (2011). Molecular mechanisms and clinical applications of angiogenesis. Nature.

[138] Zheng ZQ, Li ZX, Zhou GQ, Lin L, Zhang LL, Lv JW (2019). Long Noncoding RNA FAM225A Promotes Nasopharyngeal Carcinoma Tumorigenesis and Metastasis by Acting as ceRNA to Sponge miR-590-3p/miR-1275 and Upregulate ITGB3. Cancer Res.

[139] Wang J, Ding W, Xu Y, Tao E, Mo M, Xu W (2020). Long non-coding RNA RHPN1-AS1 promotes tumorigenesis and metastasis of ovarian cancer by acting as a ceRNA against miR-596 and upregulating LETM1. Aging (Albany NY).

[140] Zuo X, Chen Z, Gao W, Zhang Y, Wang J, Wang J (2020). M6A-mediated upregulation of LINC00958 increases lipogenesis and acts as a nanotherapeutic target in hepatocellular carcinoma. J Hematol Oncol.

[141] Yang D, Qiao J, Wang G, Lan Y, Li G, Guo X (2018). N6-Methyladenosine modification of lincRNA 1281 is critically required for mESC differentiation potential. Nucleic Acids Res.

[142] Moindrot B, Cerase A, Coker H, Masui O, Grijzenhout A, Pintacuda G (2015). A Pooled shRNA Screen Identifies Rbm15, Spen, and Wtap as Factors Required for Xist RNA-Mediated Silencing. Cell Rep.

[143] Patil DP, Chen CK, Pickering BF, Chow A, Jackson C, Guttman M (2016). m(6)A RNA methylation promotes XIST-mediated transcriptional repression. Nature.

[144] Li X, Yang L, Chen LL (2018). The Biogenesis, Functions, and Challenges of Circular RNAs. Mol Cell.

[145] Zhao X, Cai Y, Xu J (2019). Circular RNAs: Biogenesis, Mechanism, and Function in Human Cancers. Int J Mol Sci.

[146] Tang C, Xie Y, Yu T, Liu N, Wang Z, Woolsey RJ (2020). m6A-dependent biogenesis of circular RNAs in male germ cells. Cell Res.

[147] Di Timoteo G, Dattilo D, Centrón-Broco A, Colantoni A, Guarnacci M, Rossi F (2020). Modulation of circRNA Metabolism by m6A Modification. Cell Rep.

[148] Chen C, Yuan W, Zhou Q, Shao B, Guo Y, Wang W (2021). N6-methyladenosine-induced circ1662 promotes metastasis of colorectal cancer by accelerating YAP1 nuclear localization. Theranostics.

[149] Chen RX, Chen X, Xia LP, Zhang JX, Pan ZZ, Ma XD (2019). N6-methyladenosine modification of circNSUN2 facilitates cytoplasmic export and stabilizes HMGA2 to promote colorectal liver metastasis. Nat Commun.

[150] Li X, Tian G, Wu J (2021). Novel circGFRα1 Promotes Self-Renewal of Female Germline Stem Cells Mediated by m6A Writer METTL14. Front Cell Dev Biol.

[151] Legnini I, Di Timoteo G, Rossi F, Morlando M, Briganti F, Sthandier O (2017). Circ-ZNF609 Is a Circular RNA that Can Be Translated and Functions in Myogenesis. Mol Cell.

[152] Park OH, Ha H, Lee Y, Boo SH, Kwon DH, Song HK (2019). Endoribonucleolytic Cleavage of m6A-Containing RNAs by RNase P/MRP Complex. Mol Cell.

[153] Su H, Wang G, Wu L, Ma X, Ying K, Zhang R (2020). Transcriptome-wide map of m6A circRNAs identified in a rat model of hypoxia mediated pulmonary hypertension. BMC Genomics.

[154] Wang Q, Zhang H, Chen Q, Wan Z, Gao X, Qian W (2019). Identification of METTL14 in Kidney Renal Clear Cell Carcinoma Using Bioinformatics Analysis. Dis Markers.

[155] Chen Z, Ling K, Zhu Y, Deng L, Li Y, Liang Z (2021). circ0000069 promotes cervical cancer cell proliferation and migration by inhibiting miR-4426. Biochem Biophys Res Commun.

[156] Li Z, Yang HY, Dai XY, Zhang X, Huang YZ, Shi L (2021). CircMETTL3, upregulated in a m6A-dependent manner, promotes breast cancer progression. Int J Biol Sci.

[157] Zhang X, Xu Y, Qian Z, Zheng W, Wu Q, Chen Y (2018). circRNA_104075 stimulates YAP-dependent tumorigenesis through the regulation of HNF4a and may serve as a diagnostic marker in hepatocellular carcinoma. Cell Death Dis.

[158] Memczak S, Jens M, Elefsinioti A, Torti F, Krueger J, Rybak A (2013). Circular RNAs are a large class of animal RNAs with regulatory potency. Nature.

[159] Yang Y, Fan X, Mao M, Song X, Wu P, Zhang Y (2017). Extensive translation of circular RNAs driven by N6-methyladenosine. Cell Res.

[160] Yang Y, Gao X, Zhang M, Yan S, Sun C, Xiao F (2018). Novel Role of FBXW7 Circular RNA in Repressing Glioma Tumorigenesis. J Natl Cancer Inst.

[161] Wc L, Cw W, Pp L, M S, Y C, St R, et al. Translation of the circular RNA circβ-catenin promotes liver cancer cell growth through activation of the Wnt pathway. Genome biology. 2019;20(1). Available from: https://pubmed.ncbi.nlm.nih.gov/31027518/. [Cited 2023 Mar 30]. 10.1186/s13059-019-1685-4 PMC6486691 31027518

[162] Rao X, Lai L, Li X, Wang L, Li A, Yang Q (2021). N6 -methyladenosine modification of circular RNA circ-ARL3 facilitates Hepatitis B virus-associated hepatocellular carcinoma via sponging miR-1305. IUBMB Life.

[163] Zhao J, Lee EE, Kim J, Yang R, Chamseddin B, Ni C (2019). Transforming activity of an oncoprotein-encoding circular RNA from human papillomavirus. Nat Commun.

[164] Pinto R, Vågbø CB, Jakobsson ME, Kim Y, Baltissen MP, O’Donohue MF (2020). The human methyltransferase ZCCHC4 catalyses N6-methyladenosine modification of 28S ribosomal RNA. Nucleic Acids Res.

[165] Rong B, Zhang Q, Wan J, Xing S, Dai R, Li Y (2020). Ribosome 18S m6A Methyltransferase METTL5 Promotes Translation Initiation and Breast Cancer Cell Growth. Cell Rep.

[166] Goh YT, Koh CWQ, Sim DY, Roca X, Goh WSS (2020). METTL4 catalyzes m6Am methylation in U2 snRNA to regulate pre-mRNA splicing. Nucleic Acids Res.

[167] Penny GD, Kay GF, Sheardown SA, Rastan S, Brockdorff N (1996). Requirement for Xist in X chromosome inactivation. Nature.

[168] Du M, Zhang Y, Mao Y, Mou J, Zhao J, Xue Q (2017). MiR-33a suppresses proliferation of NSCLC cells via targeting METTL3 mRNA. Biochem Biophys Res Commun.

[169] Cui X, Wang Z, Li J, Zhu J, Ren Z, Zhang D (2020). Cross talk between RNA N6-methyladenosine methyltransferase-like 3 and miR-186 regulates hepatoblastoma progression through Wnt/β-catenin signalling pathway. Cell Prolif.

[170] Luo Y, Sun R, Zhang J, Sun T, Liu X, Yang B (2015). miR-506 inhibits the proliferation and invasion by targeting IGF2BP1 in glioblastoma. Am J Transl Res.

[171] Wang RJ, Li JW, Bao BH, Wu HC, Du ZH, Su JL (2015). MicroRNA-873 (miRNA-873) inhibits glioblastoma tumorigenesis and metastasis by suppressing the expression of IGF2BP1. J Biol Chem.

[172] Du C, Lv C, Feng Y, Yu S (2020). Activation of the KDM5A/miRNA-495/YTHDF2/m6A-MOB3B axis facilitates prostate cancer progression. J Exp Clin Cancer Res.

[173] MiR-451a promotes cell growth, migration and EMT in osteosarcoma by regulating YTHDC1-mediated m6A methylation to activate the AKT/mTOR signaling pathway. J Bone Oncol. 2022;33:100412. 10.1016/j.jbo.2022.100412 PMC8842083 35198364

[174] Zhu L, Zhu Y, Han S, Chen M, Song P, Dai D (2019). Impaired autophagic degradation of lncRNA ARHGAP5-AS1 promotes chemoresistance in gastric cancer. Cell Death Dis.

[175] Wang S, Wang Y, Zhang Z, Zhu C, Wang C, Yu F (2021). Long Non-Coding RNA NRON promotes Tumor Proliferation by regulating ALKBH5 and Nanog in Gastric Cancer. J Cancer.

[176] Gu Y, Niu S, Wang Y, Duan L, Pan Y, Tong Z (2021). DMDRMR-Mediated Regulation of m6A-Modified CDK4 by m6A Reader IGF2BP3 Drives ccRCC Progression. Cancer Res.

[177] Wang X, Li X, Zhou Y, Huang X, Jiang X (2022). Long non-coding RNA OIP5-AS1 inhibition upregulates microRNA-129-5p to repress resistance to temozolomide in glioblastoma cells via downregulating IGF2BP2. Cell Biol Toxicol.

[178] Xie F, Huang C, Liu F, Zhang H, Xiao X, Sun J (2021). CircPTPRA blocks the recognition of RNA N6-methyladenosine through interacting with IGF2BP1 to suppress bladder cancer progression. Mol Cancer.

[179] Lin X, Chai G, Wu Y, Li J, Chen F, Liu J (2019). RNA m6A methylation regulates the epithelial mesenchymal transition of cancer cells and translation of Snail. Nat Commun.

[180] Xi Z, Xue Y, Zheng J, Liu X, Ma J, Liu Y (2016). WTAP Expression Predicts Poor Prognosis in Malignant Glioma Patients. J Mol Neurosci.

[181] Visvanathan A, Patil V, Arora A, Hegde AS, Arivazhagan A, Santosh V (2018). Essential role of METTL3-mediated m6A modification in glioma stem-like cells maintenance and radioresistance. Oncogene.

[182] Wang Y, Zeng L, Liang C, Zan R, Ji W, Zhang Z (2019). Integrated analysis of transcriptome-wide m6A methylome of osteosarcoma stem cells enriched by chemotherapy. Epigenomics.

[183] Zhou S, Bai ZL, Xia D, Zhao ZJ, Zhao R, Wang YY (2018). FTO regulates the chemo-radiotherapy resistance of cervical squamous cell carcinoma (CSCC) by targeting β-catenin through mRNA demethylation. Mol Carcinog.

[184] Yan F, Al-Kali A, Zhang Z, Liu J, Pang J, Zhao N (2018). A dynamic N6-methyladenosine methylome regulates intrinsic and acquired resistance to tyrosine kinase inhibitors. Cell Res.

[185] Shriwas O, Priyadarshini M, Samal SK, Rath R, Panda S, Das Majumdar SK (2020). DDX3 modulates cisplatin resistance in OSCC through ALKBH5-mediated m6A-demethylation of FOXM1 and NANOG. Apoptosis.

[186] Zhang Y, Kang M, Zhang B, Meng F, Song J, Kaneko H (2019). m6A modification-mediated CBX8 induction regulates stemness and chemosensitivity of colon cancer via upregulation of LGR5. Mol Cancer.

[187] Paramasivam A, Vijayashree PJ (2020). Novel insights into m6A modification in circular RNA and implications for immunity. Cell Mol Immunol.

[188] Qian C, Cao X (2018). Dendritic cells in the regulation of immunity and inflammation. Semin Immunol.

[189] Han D, Liu J, Chen C, Dong L, Liu Y, Chang R (2019). Anti-tumour immunity controlled through mRNA m6A methylation and YTHDF1 in dendritic cells. Nature.

[190] Wang H, Hu X, Huang M, Liu J, Gu Y, Ma L (2019). Mettl3-mediated mRNA m6A methylation promotes dendritic cell activation. Nat Commun.

[191] Schmid P, Adams S, Rugo HS, Schneeweiss A, Barrios CH, Iwata H (2018). Atezolizumab and Nab-Paclitaxel in Advanced Triple-Negative Breast Cancer. N Engl J Med.

[192] Ai Y, Liu S, Luo H, Wu S, Wei H, Tang Z (2021). METTL3 intensifies the progress of oral squamous cell carcinoma via Modulating the m6A Amount of PRMT5 and PD-L1. J Immunol Res.

[193] Liu Z, Wang T, She Y, Wu K, Gu S, Li L (2021). N6-methyladenosine-modified circIGF2BP3 inhibits CD8+ T-cell responses to facilitate tumor immune evasion by promoting the deubiquitination of PD-L1 in non-small cell lung cancer. Mol Cancer.

[194] Yang S, Wei J, Cui YH, Park G, Shah P, Deng Y (2019). m6A mRNA demethylase FTO regulates melanoma tumorigenicity and response to anti-PD-1 blockade. Nat Commun.

[195] Tsuruta N, Tsuchihashi K, Ohmura H, Yamaguchi K, Ito M, Ariyama H (2020). RNA N6-methyladenosine demethylase FTO regulates PD-L1 expression in colon cancer cells. Biochem Biophys Res Commun.

[196] Su R, Dong L, Li Y, Gao M, Han L, Wunderlich M (2020). Targeting FTO Suppresses Cancer Stem Cell Maintenance and Immune Evasion. Cancer Cell.

[197] Liu Y, Liang G, Xu H, Dong W, Dong Z, Qiu Z (2021). Tumors exploit FTO-mediated regulation of glycolytic metabolism to evade immune surveillance. Cell Metab.

[198] Qiu X, Yang S, Wang S, Wu J, Zheng B, Wang K (2021). M6A Demethylase ALKBH5 Regulates PD-L1 Expression and Tumor Immunoenvironment in Intrahepatic Cholangiocarcinoma. Cancer Res.

[199] Tsuchiya K, Yoshimura K, Inoue Y, Iwashita Y, Yamada H, Kawase A (2021). YTHDF1 and YTHDF2 are associated with better patient survival and an inflamed tumor-immune microenvironment in non-small-cell lung cancer. Oncoimmunology.

